# Gene Expression Pattern and Protein Localization of Arabidopsis Phospholipase D Alpha 1 Revealed by Advanced Light-Sheet and Super-Resolution Microscopy

**DOI:** 10.3389/fpls.2018.00371

**Published:** 2018-03-21

**Authors:** Dominik Novák, Pavol Vadovič, Miroslav Ovečka, Olga Šamajová, George Komis, Jean Colcombet, Jozef Šamaj

**Affiliations:** ^1^Department of Cell Biology, Centre of the Region Haná for Biotechnological and Agricultural Research, Palacký University Olomouc, Olomouc, Czechia; ^2^UMR9213 Institut des Sciences des Plantes de Paris Saclay, Orsay, France

**Keywords:** *Arabidopsis thaliana*, development, localization, light-sheet fluorescence microscopy, microtubules, phospholipase D, At3g15730

## Abstract

Phospholipase D alpha 1 (PLDα1, At3g15730) and its product phosphatidic acid (PA) are involved in a variety of cellular and physiological processes, such as cytoskeletal remodeling, regulation of stomatal closure and opening, as well as biotic and abiotic stress signaling. Here we aimed to study developmental expression patterns and subcellular localization of PLDα1 in Arabidopsis using advanced microscopy methods such as light-sheet fluorescence microscopy (LSFM) and structured illumination microscopy (SIM). We complemented two knockout *pldα1* mutants with a YFP-tagged PLDα1 expressed under the *PLDα1* native promoter in order to study developmental expression pattern and subcellular localization of PLDα1 in *Arabidopsis thaliana* under natural conditions. Imaging of tissue-specific and developmentally-regulated localization of YFP-tagged PLDα1 by LSFM in roots of growing seedlings showed accumulation of PLDα1-YFP in the root cap and the rhizodermis. Expression of PLDα1-YFP in the rhizodermis was considerably higher in trichoblasts before and during root hair formation and growth. Thus, PLDα1-YFP accumulated in emerging root hairs and in the tips of growing root hairs. PLDα1-YFP showed cytoplasmic subcellular localization in root cap cells and in cells of the root transition zone. In aerial parts of plants PLDα1-YFP was also localized in the cytoplasm showing enhanced accumulation in the cortical cytoplasmic layer of epidermal non-dividing cells of hypocotyls, leaves, and leaf petioles. However, in dividing cells of root apical meristem and leaf petiole epidermis PLDα1-YFP was enriched in mitotic spindles and phragmoplasts, as revealed by co-visualization with microtubules. Finally, super-resolution SIM imaging revealed association of PLDα1-YFP with both microtubules and clathrin-coated vesicles (CCVs) and pits (CCPs). In conclusion, this study shows the developmentally-controlled expression and subcellular localization of PLDα1 in dividing and non-dividing Arabidopsis cells.

## Introduction

The major function of the phospholipase D (PLD) enzymes is to hydrolyse phospholipids such as phosphatidylcholine, resulting in the production of phosphatidic acid (PA) by transphosphatidylation of water and a free soluble head group, e.g., choline (Munnik and Musgrave, 2001). The *PLD* gene family shows significant expansion in plants, and it is represented by 12 *PLD* genes in *Arabidopsis thaliana*, by comparison to just two in animals and one in yeast (Wang et al., 2012). Plant PLDs are distributed in six subclasses: α-(with 3 members), β-(with 2 members), γ-(with 3 members), δ-, ε-, ζ-(all with 2 members), depending on the protein sequences and enzymatic properties of individual members (Qin and Wang, 2002; Wang, 2005; Bargmann and Munnik, 2006; Hong et al., 2016). All 12 phospholipase D (PLD) isoforms in Arabidopsis catalyze the transphosphatidylation of water, thus generating phosphatidic acid having variable signaling roles.

Previously, some PLDs were proposed to play a morphogenetic role linked to microtubules. The relation of PLDs with microtubule organization was directly shown in the case of a tobacco PLD isoform that specifically decorates cortical microtubules (Gardiner et al., 2001) leading to the assumption that PLDs may be a specific component linking cortical microtubules to the cell wall—plasma membrane—cortical cytoskeleton continuum. Later studies on transgenic Arabidopsis cell lines using pull-down assay with GFP tagged PLDδ identified PLDδ as a cortical microtubule-binding protein (Andreeva et al., 2009; Ho et al., 2009; Hong et al., 2016). Such regulatory interactions among microtubules and PLD isoforms (particularly the Arabidopsis PLDα1 isoform) proved to have functional consequences during plant responses to salt and hyperosmotic stress (Zhang et al., 2012), ABA-induced stomatal closure (Jiang et al., 2014), and drug-induced microtubule reorganization (Zhang et al., 2017a). One prominent target of PLDα1-produced phosphatidic acid is MAP65-1, a microtubule crosslinker which contains motifs for phosphatidic acid binding outside its carboxylterminal microtubule binding domain (Zhang et al., 2012). Interestingly, phosphatidic acid binding seems to occur at the self-association aminoterminus of MAP65-1. Known and expected new functions of PLDα1 in plants might link this enzyme to G proteins, cytoskeleton and vesicular trafficking (e.g., Choudhury and Pandey, 2016, 2017; Hong et al., 2016). However, overall regulation of developmental expression pattern in cell- and tissue-specific context and subcellular localization of PLDα1 in dividing cells is not known.

Dynamic cellular and subcellular changes can be monitored for long periods with light-sheet microscopy. Moreover, imaging can be done at near physiological conditions with minimal phototoxicity at high speed. The method is based on sample illumination with a thin layer of light, thus eliminating out-of-focus excitation and preventing photobleaching. The detection path is oriented orthogonally to the illumination while plant is growing vertically according to gravity vector (Ovečka et al., 2015). Super-resolution microscopy methods can bend or overcome diffraction limitations of conventional microscopes. One such method is structured illumination microscopy (SIM; Rego et al., 2012). SIM illuminates the sample with a light pattern which combines with diffraction orders of the emitting sample to Moiré patterns. Many such patterns are generated by rotation and phase shifting of the illumination pattern. Individual images taken through high-numerical aperture objectives are combined from each position of the light pattern to reconstruct the final image (Komis et al., 2015).

In this study, we utilized advanced microscopy method such as light-sheet microscopy for developmental imaging of PLDα1 under natural condition to explore cell-type specific expression. In addition, we provide a high-resolution subcellular localization of PLDα1 in both dividing and non-dividing Arabidopsis cells in the root meristem and leaf petioles.

## Materials and methods

### Plant material, mutant screens

Seedlings were grown vertically on half-strength MS medium (Murashige and Skoog, 1962) supplemented with 0.5% (w/v) gellan gum for 14 d in Fytotron with 21°C and a 16/8 h (light/dark) photoperiod. The illumination intensity was 150 μmol m^−2^ s^−1^. Plants 12–15 days old were transferred to soil and cultivated in Fytotron with 21°C and a 16/8 h (light/dark) photoperiod and with the illumination intensity of 150 μmol m^−2^ s^−1^.

We have used T-DNA insertion lines *pldα1-1* (SALK_067533) and *pldα1-2* (SALK_053785) described previously by Bargmann et al. (2009) and Zhang et al. (2004). To check the T-DNA insertions primers were designed by the SIGnAL iSect tool (http://signal.salk.edu/tdnaprimers.2.html), and PCR was performed using genomic DNA from seedlings. *A. thaliana* ecotype Columbia-0 (Col-0) was used as the control in the complementation assay (stomatal aperture measurement).

### Preparation of complemented PLDα1-YFP

To genetically complement mutant lines, the coding sequence of wild-type *PLDα1*(At3g15730) along with the native *PLDα1* promoter (1,944 bp upstream of the initiation codon ATG of *PLDα1*) was cloned into pGreen0229-YFP-Tnos vector using *Bam*HI-*Kpn*I restriction digest to generate *proPLDα1::PLDα1:YFP* construct. The constructs were confirmed by sequencing and transformed by floral dip method (Clough and Bent, 1998; Davis et al., 2009) to Arabidopsis Col-0 ecotype (wild type) as well as to *pldα1-1* and *pldα1-2* mutants using *Agrobacterium tumefaciens* strain GV 3101. In T1 generation we have selected three independent transgenic lines with the same fluorescent properties. One line was chosen and T2 or T3 progeny of BASTA-resistant transformants, carrying a single homozygous insertion, were used for experiments.

### Preparation of transgenic line carrying PLDα1-YFP and mRFP-TUB6

Arabidopsis *pldα1-2* stably expressing *proPLDα1::PLDα1:YFP* in T2 generation were crossed with Col-0 plants stably expressing *pUBQ1:mRFP::TUB6* kindly provided by Geoffrey O. Wasteneys (Ambrose et al., 2011). F1 generation plants with PLDα1-YFP and mRFP-TUB6 expression were selected based on fluorescence signal in the epifluorescence microscope (Axio Imager.M2, Carl Zeiss, Germany).

### Immunoblotting analysis

Immunoblotting analysis was performed as described previously (Takác et al., 2017). Seedlings of 5 days old *pldα1-1* PLDα1-YFP and *pldα1-2* PLDα1-YFP complemented plants, a progeny from one selected T2 plant from each independent transgenic SALK line, were used for immunoblotting analysis. Roots from ~50 seedlings of 14 days old plants of *A. thaliana*, ecotype Col-0, *pldα1-1*, and *pldα1-2* single mutants as well as *pldα1-1* PLDα1-YFP and *pldα1-2* PLDα1-YFP complemented lines were homogenized using liquid nitrogen to fine powder and the proteins were extracted in E-buffer [50 mM HEPES (pH 7.5), 75 mM NaCl, 1 mM EGTA, 1 mM MgCl_2_, 1 mM NaF, 10% (v/v) glycerol, Complete™ EDTA-free protease inhibitor and PhosSTOP™ phosphatase inhibitor cocktails (both from Roche, Basel, Switzerland]. After centrifugation at 13000 g in 4°C for 15 min, supernatants were mixed with 4-fold concentrated Laemmli buffer [final concentration 62.5 mM Tris-HCl (pH 6.8), 2% (w/v) SDS, 10% (v/v) glycerol, 300 mM 2-mercaptoethanol] and boiled for 5 min. Protein extracts were separated on 12% TGX Stain-Free™ (Bio-Rad) gels (Biorad). Equal protein amounts were loaded for each sample. Proteins were transferred to polyvinylidene difluoride (PVDF) membranes in a wet tank unit (Bio-Rad) overnight at 24 V and 4°C using the Tris-glycin-methanol transfer buffer. Membranes were blocked in a mixture of 4% (w/v) low-fat dry milk and 4% (w/v) bovine serum albumin in Tris-buffered-saline (TBS, 100 mM Tris-HCl; 150 mM NaCl; pH 7.4) at 4°C overnight. Following washing step with TBS-T (TBS, 0.1% Tween 20) membranes were incubated with polyclonal anti-phospholipase D alpha 1/2 antibody (Agrisera, Sweden) diluted 1:5000 in TBS-T containing 1% (w/v) BSA or with anti-GFP monoclonal antibody (Sigma-Aldrich, Merck, USA) diluted 1:1000 in TBS-T containing 1% (w/v) BSA at room temperature for 1.5 h. As a loading and protein transfer control, membranes were incubated with anti-beta-tubulin monoclonal antibody (Sigma-Aldrich, Merck, USA) diluted 1:2000 in TBS-T containing 1% (w/v) BSA at room temperature for 1.5 h. Following five washing steps in TBST, membranes were incubated 1.5 h at RT with a horseradish peroxidase (HRP) conjugated goat anti-rabbit IgG secondary antibody (diluted 1:5000) in the case of anti-phospholipase D alpha 1/2 primary antibody, and with a HRP conjugated goat anti-mouse IgG secondary antibody (diluted 1:5000; both from Santa Cruz Biotechnology, Santa Cruz, CA, USA) in the case of anti-GFP and anti-beta-tubulin primary monoclonal antibody. After washing in TBS-T, the signals were developed using Clarity Western ECL substrate (Biorad, Hercules, CA, USA). Luminescence was detected on Chemidoc MP documentation system (Biorad). Three biological replicates of immunoblot experiment were performed.

### Stomatal aperture measurement

Cotyledons of 7 days-old plants of various genotypes were used for stomatal closure analysis as described previously (Jiang et al., 2014). Dissected cotyledons were floated on stomatal opening buffer [10 mM 2-(N-morpholino) ethanesulfonic acid (MES-KOH), pH = 6.15 and 30 mM KCl] under light for 2 h to fully open the stomata. Then they were treated with ABA (10 μM) in stomatal opening buffer for indicated period of time. ABA stock solution was prepared in ethanol. Ethanol in stomatal opening buffer was used as a negative control. Final concentration of ethanol in experimental solutions did not exceeded 0.01% (v/v). Stomatal aperture was documented using epifluorescence microscope AxioImager.M2 (Carl Zeiss, Germany) equipped with EC Plan-Neofluar 10x/0.30 objective (Carl Zeiss, Germany) using transmission light in single focal plane and quantitatively analyzed using Fiji (ImageJ) software.

### Whole mount immunofluorescence labeling

Immunolocalization of microtubules, PLDα1, PLDα1-YFP, and clathrin in root wholemounts was done as described previously (Šamajová et al., 2014). Samples were immunolabeled with rat anti-α-tubulin (clone YOL1/34; ABD Serotec), rabbit anti-phospholipase D alpha 1/2 (Agrisera, Sweden), mouse monoclonal anti-clathrin LC (Sigma-Aldrich) or mouse anti-GFP (Abcam) primary antibodies diluted 1:300, 1:300, 1:300 and 1:100, respectively, in 3% (w/v) BSA in PBS at 4°C overnight. In the case of double or triple co-immunolocalization a sequential immunolabeling was performed. Secondary antibodies included Alexa-Fluor 488 goat anti-rat, Alexa-Fluor 488 goat anti-mouse or Alexa-Fluor 546 goat anti-rat IgGs were diluted 1:500 in PBS containing 3% (w/v) BSA for 3 h (1.5 h at 37°C and 1.5 h at room temperature). Where necessary, nuclei were counterstained with DAPI. Microscopic analysis of immunolabeled samples was performed with a Zeiss 710 CLSM platform (Carl Zeiss, Jena, Germany), using excitation lines at 405, 488, and 561 nm from argon, HeNe, diode, and diode pumped solid-state lasers.

### Light-sheet fluorescence microscopy

Developmental live cell imaging of 2–3 days old Arabidopsis plants with PLDα1-YFP expression was done with the light-sheet Z.1 fluorescence microscope (Carl Zeiss, Germany) equipped with W Plan-Apochromat 20x/1.0 NA or W Plan-Apochromat 40x/1.0 NA objectives (Carl Zeiss, Germany) and two LSFM 10x/0.2 NA illumination objectives (Carl Zeiss, Germany). Seedlings were prepared in fluorinated ethylene propylene (FEP) tubes with an inner diameter of 2.8 mm and wall thickness of 0.2 mm (Wolf-Technik, Germany) according to the “open system” protocol for long-term live-cell imaging of *A. thaliana* seedlings described by Ovečka et al. (2015). Root was growing in the block of the culture medium inside of the FEP tube and upper green part of the seedling developed in an open space of the FEP tube with the access to air. Sample holder with the sample was placed into observation chamber of the light-sheet microscope tempered to 22°C using a Peltier heating/cooling system. Before insertion of the sample to the microscope plants were ejected slightly out of the FEP tube allowing imaging of the root in the block of solidified culture medium, but without the FEP tube. Before the imaging, liquid medium filling the observation chamber was filter-sterilized using a sterile syringe filter. Roots were imaged using dual-side light-sheet illumination with excitation laser line 514 nm, beam splitter LP 580 and with emission filter BP525-565. Images were recorded with the PCO.Edge sCMOS camera (PCO AG, Germany) with the exposure time 30 ms and the imaging frequency of every 5 min in Z-stack mode for 5–20 h. Scaling of recorded images in x, y, and z dimensions was 0.228 × 0.228 × 0.499 μm. For counterstaining of plant cell walls with propidium iodide, seedlings were germinating and growing in blocks of the solidified culture medium containing 1 μg.ml^−1^ of propidium iodide (Invitrogen, USA) before imaging. Seedlings (2 days old) growing directly in the solidified medium containing propidium iodide were transferred to the light-sheet microscope for root imaging.

### Spinning disk and confocal laser scanning microscopy

Hypocotyls, leaves with pavement cells, stomata, and trichomes of 5–8 DAG Arabidopsis plants with PLDα1-YFP expression were documented with spinning disk microscope (Cell Observer SD, Carl Zeiss, Germany) equipped with Plan-Apochromat 20x/0.8 (Carl Zeiss, Germany) and Plan-Apochromat 63x/1.40 Oil (Carl Zeiss, Germany) objectives. Cells were imaged with excitation laser 514 nm and with emission filter BP535/30 for YFP. Cotyledons, petioles and guard cells were documented with confocal laser scanning microscope LSM 710 (Carl Zeiss, Germany) equipped with Plan-Apochromat 20x/0.8 (Carl Zeiss, Germany) and alpha Plan-Apochromat 63x/1.46 Oil (Carl Zeiss, Germany) objectives. Plants of 6 DAG were stained with 4 μM FM4-64 (Invitrogen, USA) diluted in half-strength liquid MS medium for 90 min before imaging. Samples were imaged with excitation lasers 514 nm for YFP and 561 nm for mRFP and FM4-64, beam splitters MBS 458/514 for YFP, MBS 458/561 for mRFP and MBS 488/561for FM4-64. Emission spectrum used were 519–550 nm for YFP, 590–610 nm for mRFP and 651–759 nm for FM4-64.

### Structured illumination microscopy

The same immunolabeled wholemount samples examined with CLSM were also analyzed via a Zeiss SIM platform coupled with a PCO.Edge 5.5 sCMOS camera (Elyra PS.1, Carl Zeiss, Germany). Fluorophores were excited with the 405, 488, 561, and 647 nm laser lines. For acquisition with a 63x/1.40 oil immersion objective, the grating pattern was set to 5 rotations with 5 standard phase shifts per angular position. In case of Z-stacks, Nyquist sampling was selected to be the smallest one (corresponding to DAPI channel with 91 nm section thickness), leading to oversampling of the rest of the channels. Image reconstruction was done according to previously published procedures (Komis et al., 2015).

### Image processing

The post-processing, default deconvolution using Nearest Neighbour or Constrained Iterative algorithms and profile measurement of all fluorescence images in this study, including 3D reconstruction or maximum intensity projection from individual z-stacks and creating subsets was done using ZEN 2010 software. All images exported from ZEN 2010 software were assembled and captioned in Microsoft PowerPoint to final figures.

## Results

### Expression patterns of PLDα1-YFP in Arabidopsis plants

**Figure 1:**
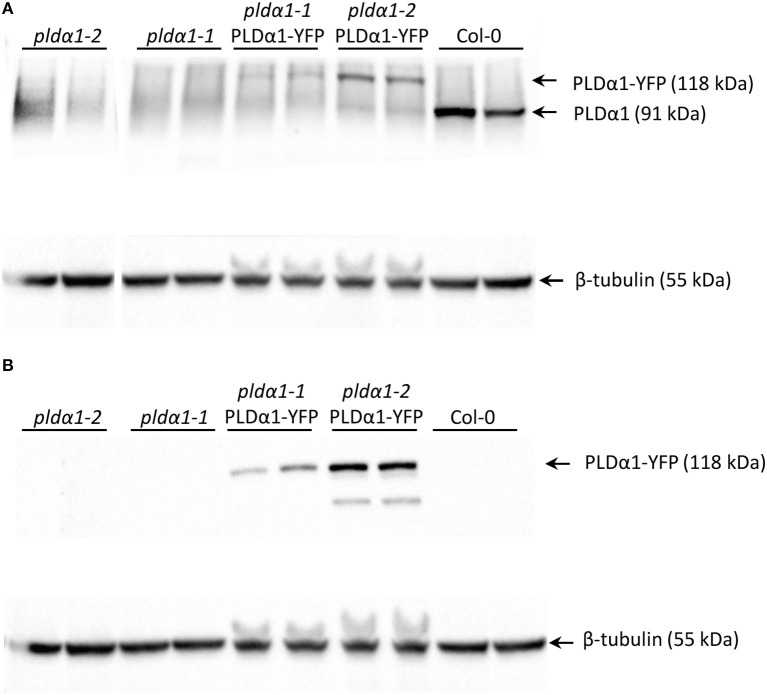
Detection of PLDα1 in roots of *pldα1* mutants and in rescued *pldα1* mutants stably transformed with *proPLDα1::PLDα1:YFP* construct. **(A)** Immunoblots of *pldα1* mutants and rescued *pldα1* mutants probed with anti-PLDα1/2 antibody. **(B)** Immunoblots of *pldα1* mutants and rescued *pldα1* mutants probed with anti GFP antibody. PVDF membranes (BioRad) were probed with anti-beta tubulin as a loading control.

In order to characterize the roles of PLDα1 in plant development, we performed *in vivo* cell- and tissue-specific expression analysis of the PLDα1-YFP driven by native *PLDα1* promoter in two stably transformed *pldα1* mutants of *A. thaliana*. Thus, both *pld*α-*1-1* and *pld*α-*1-2* mutants were stably transformed with *proPLDα1::PLDα1:YFP* construct using the floral dip method (Clough and Bent, 1998). Comparison of *proPLDα1::PLDα1:YFP* expression patterns in different aerial organs and tissues of *pldα1-1* and *pldα1-2* mutants stably expressing PLDα1-YFP is presented in Figure S1. To prove the expression of PLDα1-YFP fusion protein in experimental plants (*pldα1-1* and *pldα1-2* mutant plants stably transformed with *proPLDα1::PLDα1:YFP* construct), we performed SDS-PAGE with immunoblot analysis using anti-phospholipase D alpha 1/2 and anti-GFP antibodies. This analysis confirmed the presence of PLDα1-YFP fusion protein with the expected molecular mass of 118 kDa, using both anti-PLDα1 and anti-GFP antibodies (Figures 1A,B). In Col-0, which was used as a control, anti-PLDα1 antibody showed a protein band with a molecular mass of 91.8 kDa corresponding to PLDα1. We also confirmed the absence of PLDα1 protein in both *pldα1-1* and *pldα1-2* mutant plants. In addition, Col-0, *pldα1-1*, and *pldα1-2* mutant plants were used as negative controls for the use of the anti-GFP antibody and we did not observe any band in these lines (Figure 1B).

### Functional complementation of *pldα1-1* and *pldα1-2* mutants with PLDα1-YFP expression driven under its own promoter

**Figure 2:**
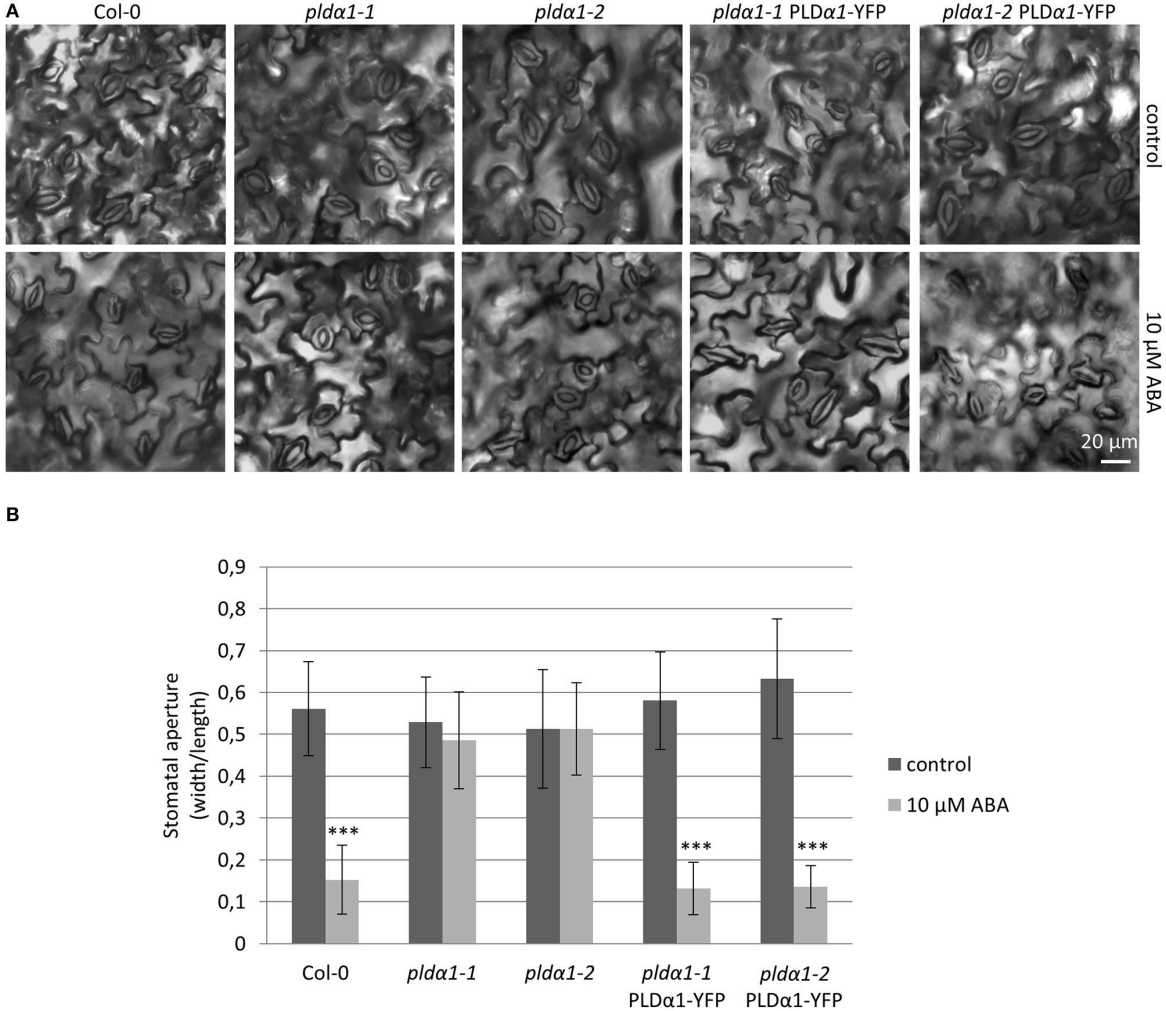
ABA-induced stomatal closure in *pldα1* mutants and rescued plants. **(A)** Cotyledon epidermis of 7 days old Col-0, *pldα1-1, pldα1-2, pldα1-1* PLDα1-YFP, and *pldα1-2* PLDα1-YFP plants showing opening of stomata in control condition and after 2 h treatment with 10 μM ABA. **(B)** Aperture of cotyledon stomata guard cells of Col-0, *pldα1-1, pldα1-2, pldα1-1* PLDα1-YFP, and *pldα1-2* PLDα1-YFP (measured as a ratio of aperture width and length, which is reduced upon its closure) in control condition and after 2 h treatment with 10 μM ABA. Bar charts represent the mean ± SD for *n* = 40. Three asterisks indicate statistically significant differences (two-tailed paired *t*-test, *P* < 0.001) in stomatal closure.

Cellular levels of abscisic acid (ABA) increase in responses to drought and salt stresses, which promotes stomatal closure in order to prevent water loss. Although PLDα1 is the most predominant PLD in plants, *pldα1* knockout mutants do not exhibit significant phenotypical changes (Fan et al., 1997; Zhang et al., 2012). Nevertheless, PLDα1 controls proper water balance in plants responding to ABA by stomatal closure and this response is impaired in the *pldα1* knockout mutants (Jiang et al., 2014; Pleskot et al., 2014; Zhang et al., 2017a). To verify that *pldα1-1* and *pldα1-2* mutants were complemented with *proPLDα1::PLDα1:YFP* construct and show wild type-like behavior, we examined stomatal closure ability of these revertants by measuring stomatal aperture in cotyledons of 7 days old plants after treatment with 10 μM ABA. We have observed significantly increased stomatal closure after ABA treatment of wild type plants in comparison to *pldα1-1* and *pldα1-2* mutants, which were ABA-insensitive and showed no change in stomatal apertures (Figure 2). These results were consistent with published data (Zhang et al., 2004; Jiang et al., 2014). Importantly, *pldα1-1* and *pldα1-2* mutant plants genetically complemented with *proPLDα1::PLDα1:YFP* construct reacted to ABA treatment by substantial decrease of stomatal aperture, similarly to the wild type plants (Figure 2). These results confirmed the phenotypical complementation of *pldα1-1* and *pldα1-2* mutants with *proPLDα1::PLDα1:YFP* construct which can be considered as a functional one for further expression and localization studies.

### Developmental expression pattern and localization of PLDα1-YFP in Arabidopsis plants

**Figure 3:**
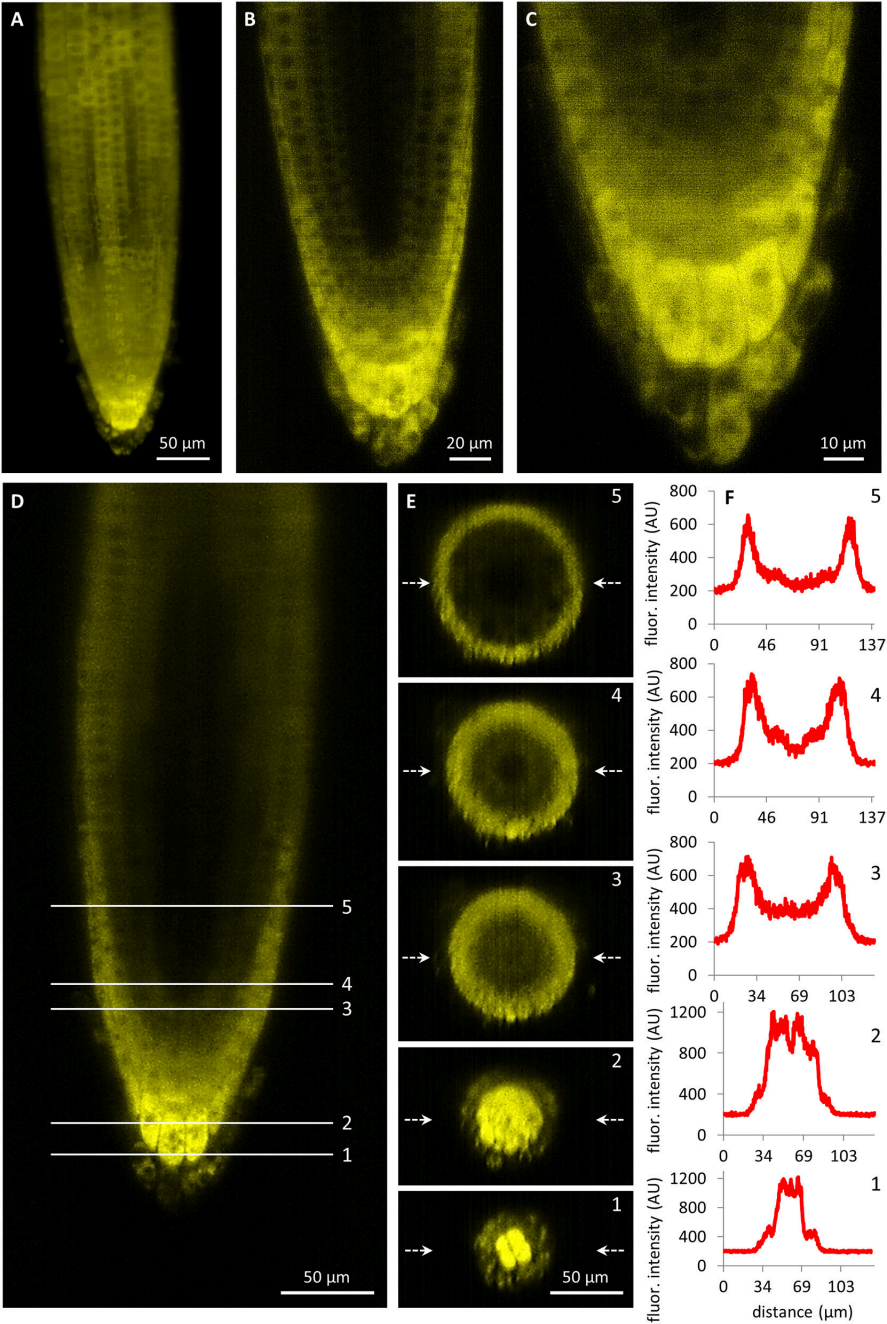
Light-sheet fluorescence microscopy localization of PLDα1-YFP driven by *PLDα1* own promoter in *Arabidopsis thaliana* roots. **(A)** Overview of PLDα1-YFP localization in different tissues of the root tip constructed from maximum intensity projection of 330 optical sections (with thickness of 0.5 μm each). The amount of PLDα1-YFP fluctuated in root rhizodermal cells while the highest localization was visible in the root cap cells. **(B)** Median optical section of the root tip revealed differential distribution of PLDα1-YFP with the strongest expression in the root cap and lateral root cap cells and with much lower production of PLDα1-YFP in rhizodermal, cortical and endodermal cell layers and with very low production in procambial cells. **(C)** Detail of the root cap showing the strongest expression level of PLDα1-YFP in central columella cells and particularly in cells of the third root cap layer. **(D–F)** Qualitative and semi-quantitative evaluation of the relative PLDα1-YFP distribution in longitudinal and radial zonation of the root tip. Five profiles at different positions of the root tip **(D)** were visualized into orthogonal projections of radial root sections **(E)** and profiles in the median positions of the radial root sections (indicated by arrows) were quantitatively displayed **(F)**. Images were taken from *pldα1-1* mutant plants stably expressing PLDα1-YFP.

Observation of developmental expression pattern and localization of PLDα1-YFP fusion protein has been done in *pldα1-1* mutant stably expressing a *proPLDα1::PLDα1:YFP* construct at cell-, tissue- and organ-specific levels using light-sheet fluorescence microscopy (LSFM). Subcellular localization was performed using confocal and spinning disk microscopy. Developmental LSFM has been performed with 2- to 3-days old seedlings that were growing inside of the microscope imaging chamber in time periods ranging from 5 to 20 h. During these imaging periods, roots of experimental plants exhibited continuous growth at constant root growth rates. LSFM offered the possibility not only to localize PLDα1-YFP at the cellular level in root surface tissues (Figure 3A), but it also allowed deep root imaging and tissue-specific visualization and localization of PLDα1-YFP in internal root tissues (Figure 3B, Figures S2, S3).

Imaging of tissue-specific expression of PLDα1-YFP in roots using LSFM revealed developmental regulation of PLDα1-YFP amount in the root apex. The expression levels of PLDα1-YFP in the root meristematic zone including rhizodermis, cortex, and procambium were relatively low (Figure 3B). On the other hand, particularly strong expression levels were revealed in the apical and lateral root cap cells (Figure 3C). Remarkably strong expression was observed in central columella cells and particularly in cells of the third columella layer (Figures 3B,C). Semi quantitative evaluation of the PLDα1-YFP amount in different cell layers of root apex (Figure 3D) revealed a steep gradient between third and fourth outermost layers of the central root cap (Figures 3D–F; profiles 1–2). There was a relatively low amount of PLDα1-YFP in the primary meristems at the position of the stem cell niche in comparison to the lateral root cap cells (Figures 3D–F; profile 3). Proximally to the region of initial cells there was a clear gradient in the PLDα1-YFP amount within the radial organization of the root meristem with the highest level in the lateral root cap cells, much lower level in the rhizodermis, cortex and endodermis, and the lowest level in central cylinder tissues (Figures 3D–F; profile 4). Different expression levels among lateral root cap cells, dermal tissues (rhizodermis, cortex, and endodermis) and central cylinder tissues were clearly visible in the central part of the root meristematic zone (Figures 3D–F; profile 5).

**Figure 4:**
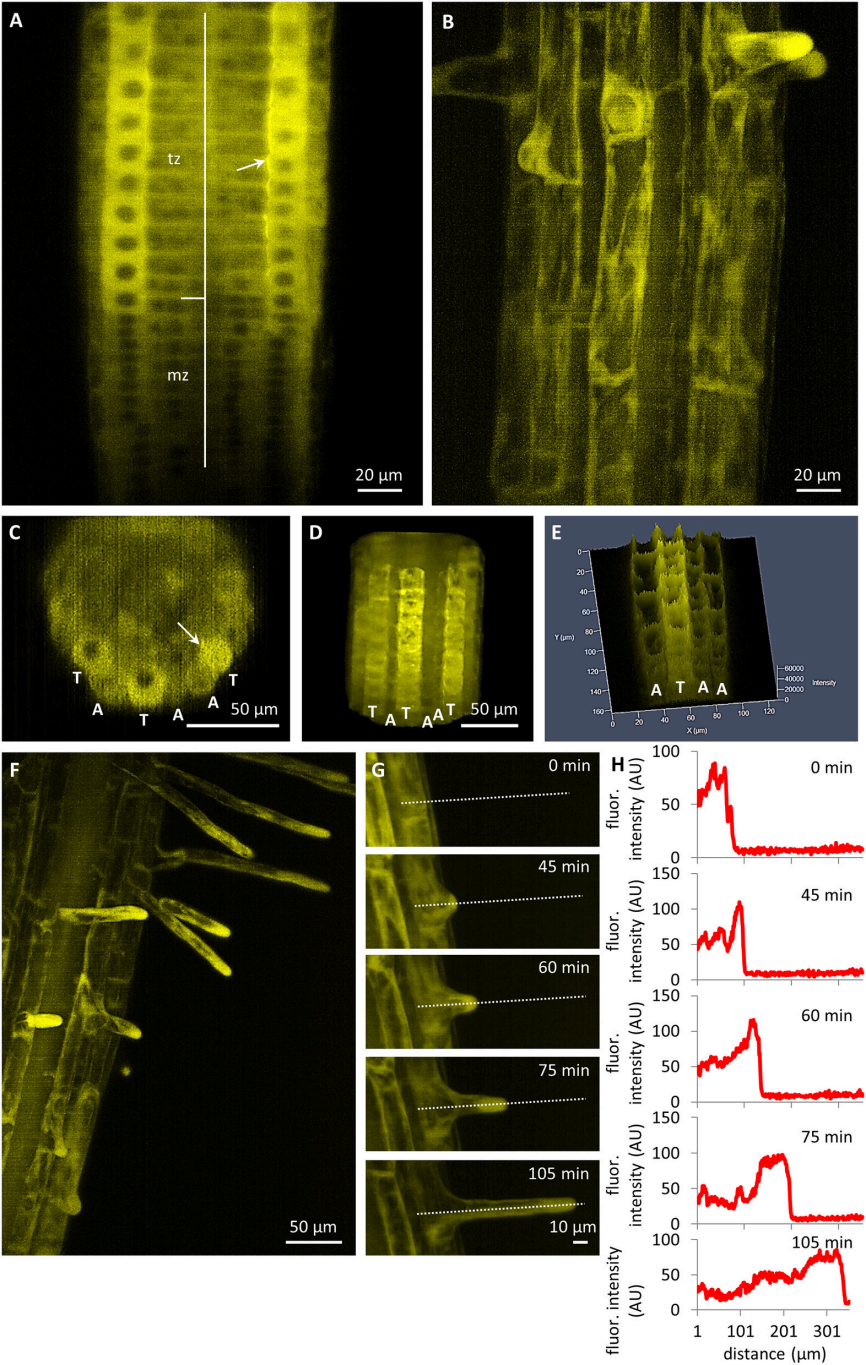
Tissue- and cell-specific localization of PLDα1-YFP driven by its own native promoter in the root of *Arabidopsis thaliana* by light-sheet fluorescence microscopy. **(A)** Distribution of PLDα1-YFP in meristematic zone (mz) with relatively low expression and in transition zone (tz) with enhanced expression. **(B)** Differentiation zone of the root with stronger expression level of PLDα1-YFP in trichoblasts and bulges of emerging root hairs and lover expression level in atrichoblast cell files. **(C–E)** Orthogonal projection **(C)**, 3D-rendering **(D)** and intensity-based 3D visualization **(E)** of root differentiation zone showing higher expression level of PLDα1-YFP in rhizodermal trichoblast cell files (labeled as T) and lover expression level in atrichoblast cell files (labeled as A). Arrows in **(A,C)** point local accumulation of PLDα1-YFP at the cell corner of the trichoblasts in the contact with two underlying cortical cells. **(F)** Enhanced localization of PLDα1-YFP in trichoblast cells at the stage of root hair formation and apparent accumulation of PLDα1-YFP in growing root hairs. **(G)** Time-course recording of accumulation and relocation of PLDα1-YFP during the root hair outgrowth in trichoblast root cell. Time frames of individual developmental stages are indicated in min. Dotted lines along the median longitudinal axis of the root hair indicate the position of fluorescence intensity profile measurement. **(H)** Fluorescence intensity profiles of PLDα1-YFP distribution corresponding to particular developmental stages of the root hair formation from the trichoblast root cell in **(G)**. Images were taken from *pldα1-1* mutant plants stably expressing PLDα1-YFP using light-sheet microscopy.

In comparison to the relatively low expression level of the PLDα1-YFP in the root meristem, a dramatic enhancement was detected in the root transition zone, particularly in the rhizodermis (Figure 4A). Rhizodermal cells showed much stronger expression levels in the trichoblast cell files compared to the atrichoblast ones (Figures 4B–E). The relatively strong expression of PLDα1-YFP in trichoblast cells of the transition root zone revealed one additional aspect of particular interest. It was the strongly polarized localization of PLDα1-YFP at the cell corner of the trichoblasts facing the cleft contact with two underlying cortical cells (Figures 4A,C). Thereon, the strong expression levels of PLDα1-YFP in trichoblast cell files was also maintained later in the development of root hairs during bulge formation (Figure 4B) and in tip-growing root hairs (Figures 4F–H). Time-course semi quantitative evaluation of PLDα1-YFP distribution clearly revealed its accumulation in growing tips of root hairs (Figures 4G,H). The PLDα1-YFP expression pattern in growing roots thus reflected the tissue-specific and developmentally-regulated transition from low PLDα1-YFP protein levels in actively dividing cells of the root apical meristem to much enhanced protein accumulation in the root transition zone harboring post-mitotic cells preparing for cell elongation (Figures 3D–F). Cell differentiation in root tissues led to localized accumulation of PLDα1-YFP, particularly in the developing rhizodermis, where PLDα1-YFP accumulated preferentially in trichoblasts (Figures 4B–E), especially during the process of root hair formation (Figures 4F–H). In all root tissues expressing moderate levels of PLDα1-YFP (root cap cells, root transition zone, trichoblast cell files and tip growing root hairs) we observed cytoplasmic localization of the fusion protein.

To better identify cell margins and cell types, roots were counterstained with propidium iodide (PI). Although seedling treatment with PI might affect rates of root growth and elongation, expression patterns of PLDα1-YFP in diverse root tissues and cell types were the same as described above (Figures S2–S5).

**Figure 5:**
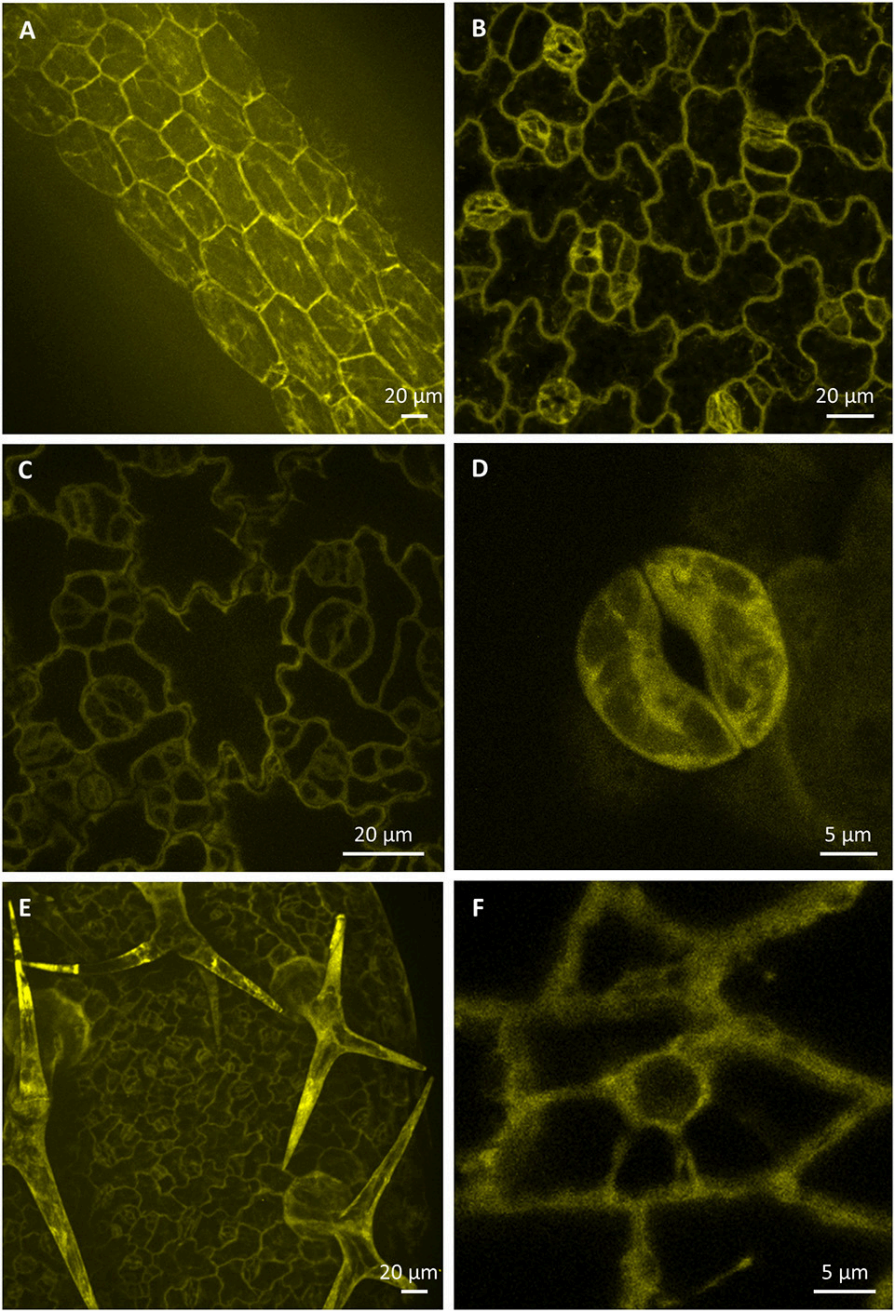
Localization of PLDα1-YFP driven by *PLDα1* own promoter in different aerial organs and tissues of *Arabidopsis thaliana* seedlings. Images were taken from different cell types of aerial tissues of living *pldα1-2* mutants stably expressing PLDα1-YFP driven by its own promoter using confocal and spinning disk microscopy. Localization of PLDα1-YFP in hypocotyl epidermal cells **(A)**, cotyledon epidermal cells and stomata **(B)**, leaf epidermal pavement cells and stomata guard cells **(C)**, leaf stoma guard cells **(D)**, leaf epidermal cells and trichomes **(E)**, and petiole epidermal cell **(F)**. Spinning disk microscopy **(A,C,D)**, confocal microscopy **(B,E,F)**.

Expression pattern and localization of PLDα1-YFP in different cell types of aerial parts of 6 days old seedlings were documented with confocal and spinning disk microscopy. Relatively high expression level of PLDα1-YFP was observed in hypocotyl epidermal cells (Figure 5A), in pavement cells and stomata guard cells of cotyledons (Figure 5B), in leaf epidermis and stomata guard cells of leaves (Figures 5C,D). Consistently with strong expression level of PLDα1-YFP in rhizodermis and in developing root hairs we observed also strong expression of PLDα1-YFP in leaf trichomes (Figure 5E). In more detail, high amounts of PLDα1-YFP were found at tips of trichome branches (Figure 5E). A high level of PLDα1-YFP was observed also in epidermal cells of leaf petioles (Figure 5F).

In cells of all examined aerial tissues, PLDα1-YFP was localized in the cytosol and predominantly at the cell cortex in the vicinity of the plasma membrane. Additionally, PLDα1-YFP was localized in cytoplasmic strands of interphase cells (Figure 5F). Immunofluorescence localization of PLDα1 protein in root meristematic cells of wild type Col-0 plants using anti-PLDα1/2 antibody confirmed its homogeneous localization in the cytosol (Figure S6).

**Figure 6:**
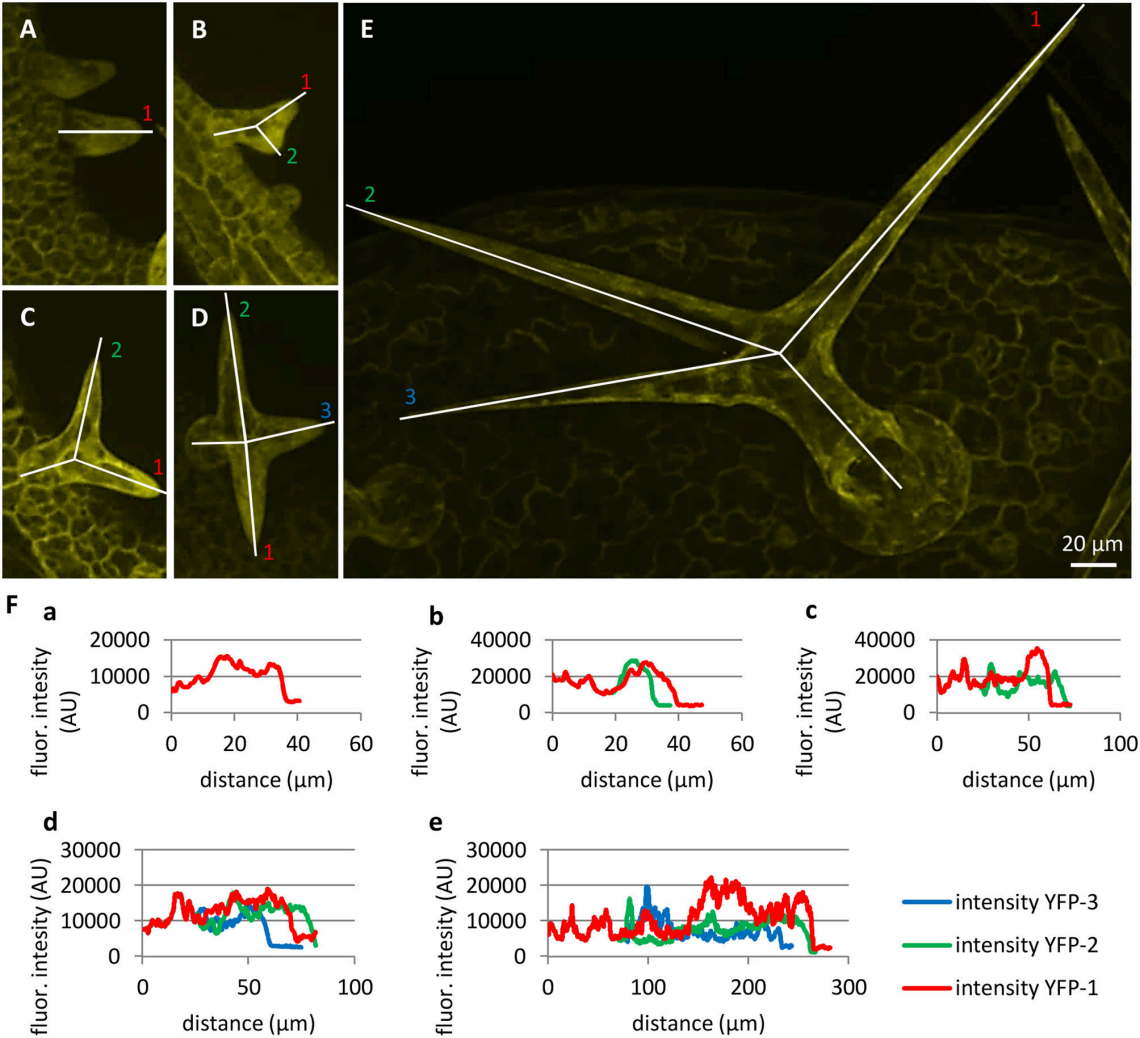
Localization and accumulation of PLDα1-YFP in developing leaf trichomes in *pldα1-2* mutant plants stably expressing PLDα1-YFP driven by *PLDα1* own promoter. Images of maximum intensity projection were reconstructed from individual optical sections taken at the same live cell imaging condition from first **(B,D,E)** and second **(A,C)** true leaves of *pldα1-2* mutants stably expressing PLDα1-YFP. Developing trichomes are displayed from early developing stages with no branches **(A)**, with progressing two branches **(B, C)**, growing three branches **(D)** to maturating three branches **(E)**. **(F)** Profiles of fluorescence intensity of YFP distribution in individual trichome branches indicated by lines in **(A–E)**.

Accumulation of PLDα1-YFP in growing root hairs (Figures 4F–H) suggested its role in actively growing cell domains. To test this possible scenario in leaf trichomes, we identified individual stages of trichome development in the first true leaf and we performed semiquantitative evaluation of PLDα1-YFP distribution along single profiles in individual trichome branches. To quantify PLDα1-YFP developmental redistribution during trichome formation, we measured profiles of PLDα1-YFP fluorescence in young trichome primordia without branches (Figure 6A), in each individual branch of growing trichomes during later developmental stages (Figures 6B–D) up to final stage of fully developed three-branched trichomes (Figure 6E). Profiling of fluorescence intensity along individual trichome branches clearly revealed higher accumulation of PLDα1-YFP at the tip of actually growing branch during trichome development (Figure 6F).

### Association of PLDα1-YFP with microtubules

**Figure 7:**
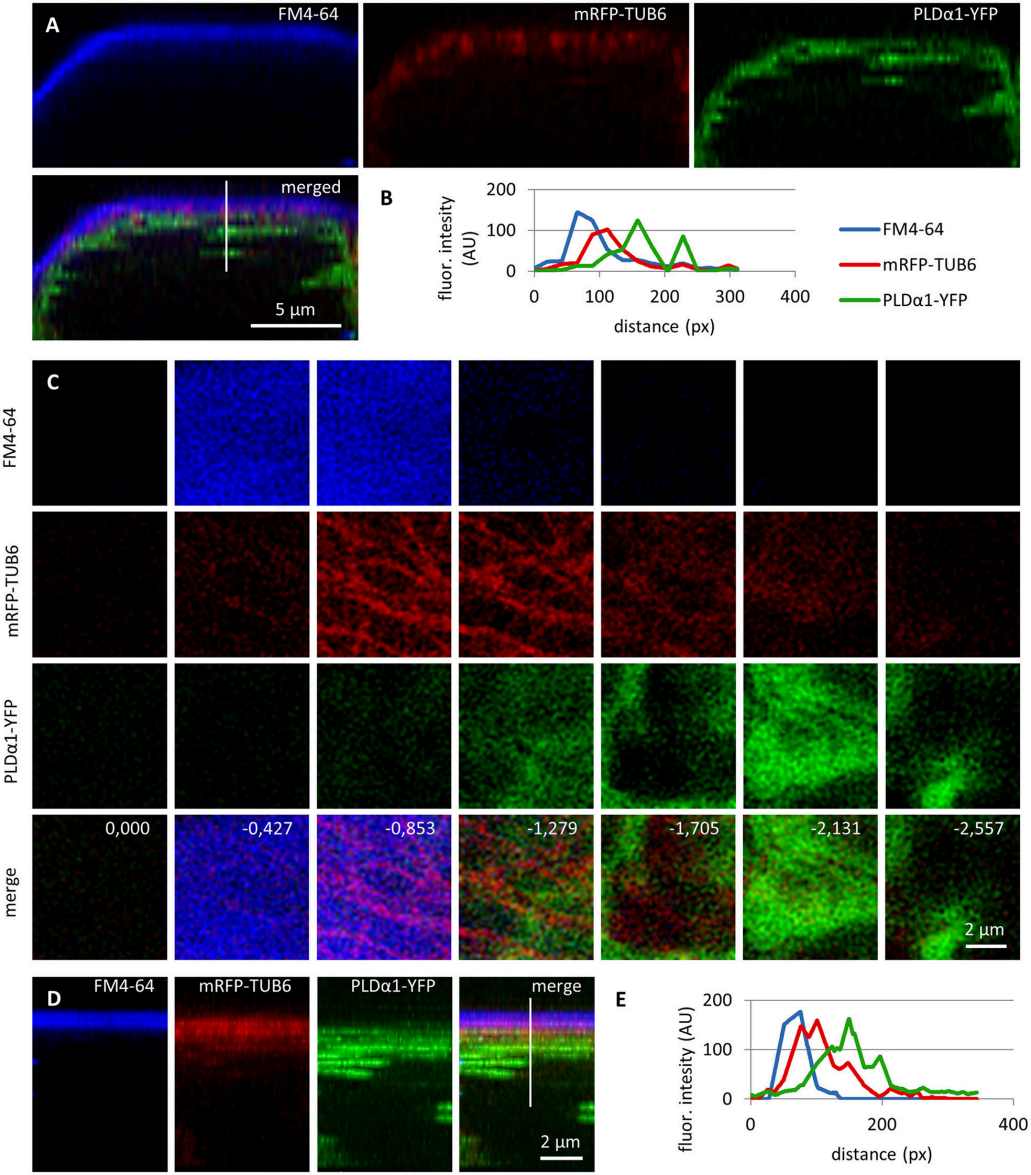
Subcellular localization of PLDα1-YFP and cortical microtubules in respect to the plasma membrane in leaf petiole epidermal cells of the *pldα1-2* mutant plants stably expressing PLDα1-YFP driven by *PLDα1* own promoter. Living interphase petiole cells with the expression of PLDα1-YFP (in green) and mRFP-TUB6 (in red) were counterstained with FM4-64 for delineation of the plasma membrane (in blue). **(A)** Orthogonal view of the 3D-reconstructed petiole cell from the z-stack imaging showing localization of plasma membrane, cortical microtubules and PLDα1. Line in merged image indicates position of measured profile. **(B)** Profile intensity of fluorescence distribution of the plasma membrane (blue), cortical microtubules (red) and PLDα1 (green) based on the distance from the cell surface. **(C)** Frontal view reconstructed from the individual z-stacks starting from the cell surface of the petiole cell to the cortical cytoplasm in steps indicating thickness of individual optical sections in μm. Individual channels are shown separately for the plasma membrane (labeled in blue), cortical microtubules (labeled in red) and PLDα1 (labeled in green), while the merge image shows the overlay of all three channels with the depth annotation. **(D)** Maximum intensity projection of the same image as in C from the side view (orthogonal projection) with the line indicating position of measured profile. **(E)** Profile intensity of fluorescence distribution of the plasma membrane (blue), cortical microtubules (red) and PLDα1 (green) from **(D)** based on the distance from the cell surface. Analysis made in confocal microscope.

In order to investigate the localization of PLDα1-YFP fusion protein in respect to cortical and mitotic microtubules we crossed *plda-1-2* mutant plants stably expressing *proPLDα1::PLDα1:YFP* construct with Col-0 plants stably expressing p*UBQ1::mRFP:TUB6* construct (red fluorescent protein marker fused to Arabidopsis alfa-tubulin 6 isoform, Ambrose et al., 2011). Labeling of the plasma membrane in cells of such crossed line was performed with FM4-64. The co-localization experiments were done in non-dividing leaf petiole epidermal cells using confocal laser scanning microscopy (Figure 7). 3-D rendering and orthogonal projections showed very close association of cortical microtubules with the plasma membrane and predominant localization of PLDα1-YFP in the cortical cytoplasm (Figure 7A). Merge image of all three markers (Figure 7A) and semi-quantitative measurement of fluorescence intensities along transversal profile in the cell cortex (Figure 7B) revealed only poor co-localization, but rather association of PLDα1-YFP with cortical microtubules. This was evident also from spatial separation of individual optical sections from 3-D scans of the cell cortex starting from the cell surface. By taking individual optical sections of 420 nm thickness (Figure 7C), we observed uppermost signal of the FM4-64 related to the plasma membrane first, followed by mRFP signal corresponding to cortical microtubules located beneath the plasma membrane, and only then first appearance of the YFP signal related to the PLDα1. In merge image, the plasma membrane signal was enriched in second and third optical section (0.000 to −0.853 μm from the cell surface), network of cortical microtubules was present in third to fifth optical section (−0.853 to −1.705 μm from the cell surface), while PLDα1-YFP signal was enriched only in fourth to sixth optical section (−1.279 to −2.131 μm from the cell surface). Association and partial colocalization of PLDα1-YFP with cortical microtubules (detected as yellow spots in merge images) is visible only on the cytoplasmic face (Figure 7C, optical section −1.279), but not on the membrane face (Figure 7C, optical section −0.853) of the cortical microtubule network. Sandwich-like arrangement of the plasma membrane, cortical microtubule network, and PLDα1-YFP was evident also from orthogonal view of the examined cell cortex area (Figure 7D), proven also by semiquantitative fluorescence profile intensity measurement (Figure 7E). These experiments revealed predominantly cytoplasmic localization of PLDα1-YFP.

### Colocalization of PLDα1-YFP with microtubules in dividing cells

**Figure 8:**
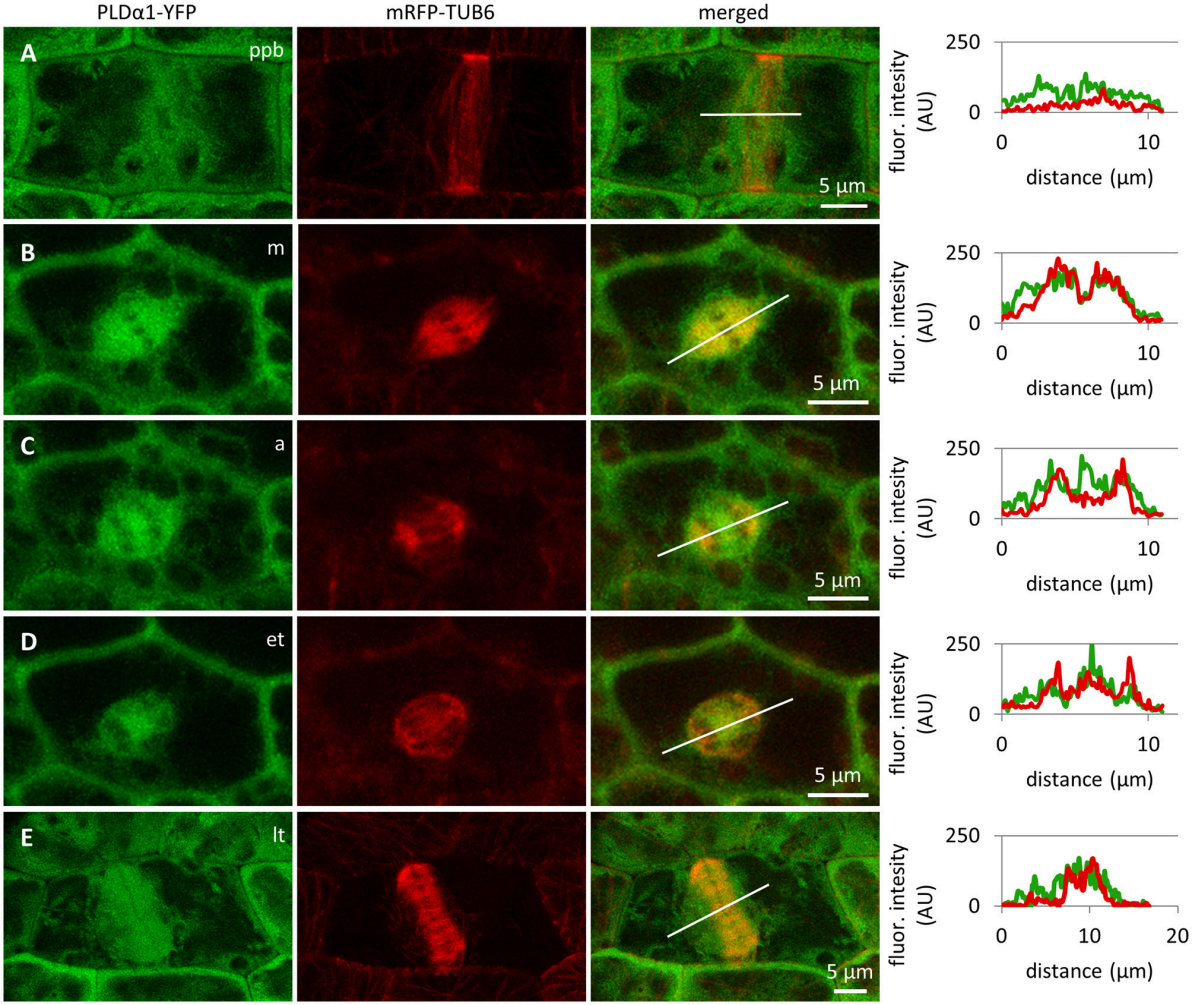
Subcellular localization of PLDα1-YFP during cell division. Live cell imaging of PLDα1-YFP (green) and mRFP-TUB6 (red) in dividing leaf petiole epidermal cell of the *pldα1-2* mutant plants stably expressing PLDα1-YFP driven by *PLDα1* own promoter. PLDα1-YFP was enriched at the location of preprophase band of microtubules (PPB) **(A)**, while it strongly associated with the mitotic spindle **(B–D)**, early **(D)**, and late **(E)** phragmoplast. Profiles of fluorescent intensity of YFP and mRFP distribution measured at individual cell division stages are indicated by lines **(A–E)**. ppb, preprophase band of microtubules; m, metaphase; a, anaphase; et, early telophase; lt, late telophase.

**Figure 9:**
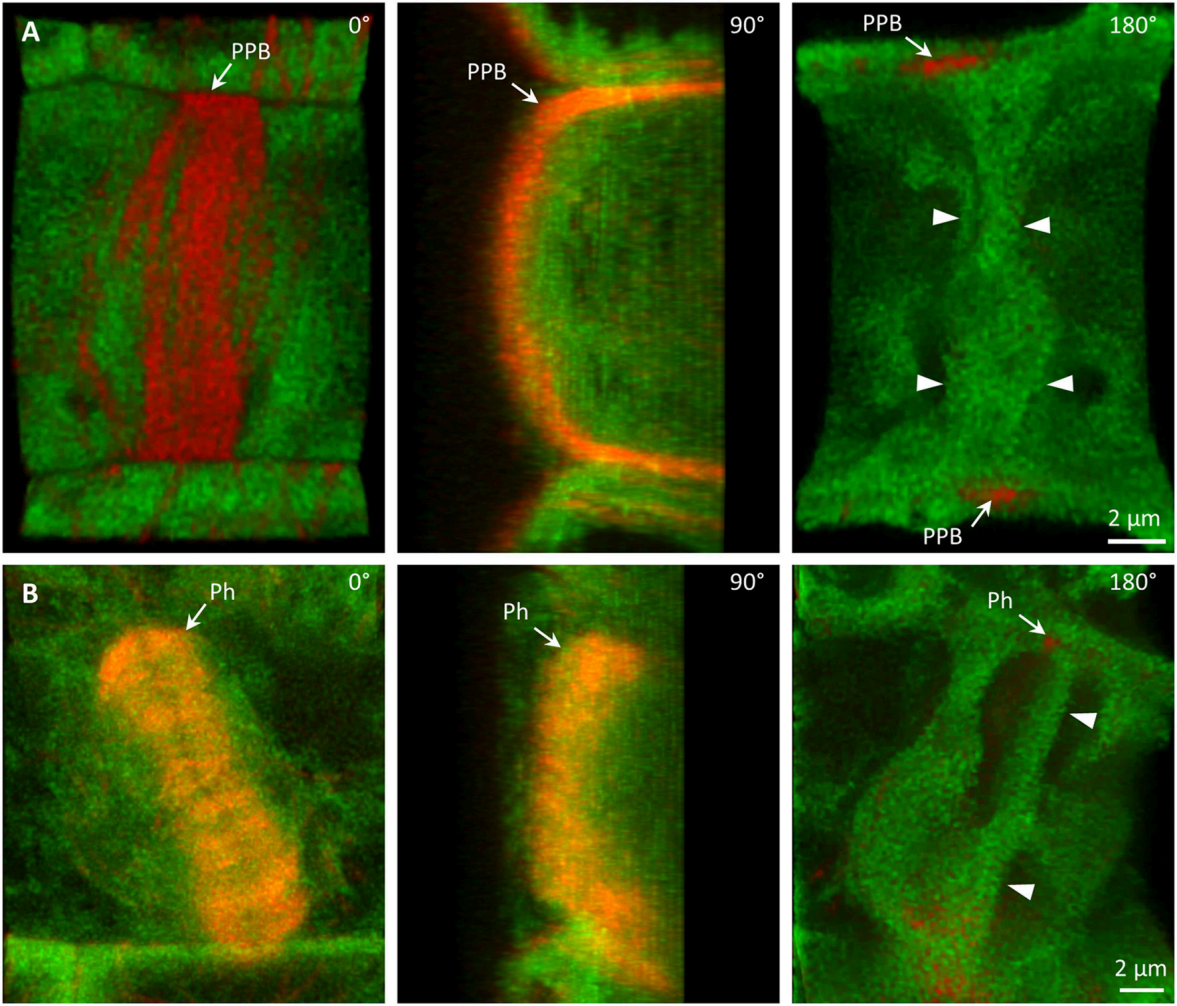
Association of PLDα1-YFP with microtubules during cell division. Live cell imaging of dividing leaf epidermal petiole cells of complemented *pldα1-2 mutant* seedlings expressing PLDα1-YFP and mRFP-TUB6. **(A)** Rotation of 3-D reconstructed pre-prophase cell with established PPB (arrows) showing localization of PLDα1-YFP in cytoplasmic disk between cell nucleus and cortical PPB zone (arrowheads). Individual positions of rotating orthogonal projection correspond to Video S1. **(B)** Rotation of 3-D reconstructed cytokinetic cell with ring phragmoplast (Ph, arrows) showing localization of PLDα1-YFP among phragmoplast microtubules, in cytoplasm around the phragmoplast and in emerging cell plate in the central zone of the ring phragmoplast (arrowheads). Individual positions of rotating orthogonal projection correspond to Video S2.

Colocalization of PLDα1-YFP with mitotic microtubule arrays was observed in dividing epidermal cells of leaf petioles using spinning disk microscopy (Figures 8A–E). Association of PLDα1-YFP with the pre-prophase band of microtubules (PPB) was evident in the pre-prophase and prophase stage (Figure 8A), with mitotic spindle during metaphase to anaphase (Figures 8B–D) and with progressing phragmoplast during cytokinesis (Figure 8E). In the pre-prophase and prophase stage PLDα1-YFP accumulated in the cell cortex in a ring-like structure that was broader as PPB. This indicates that PLDα1-YFP, in addition to its colocalization with microtubules inside the PPB, also surrounded PPB in the cortical cytoplasm (Figures 8A, 9A). Additionally, PLDα1-YFP was enriched also in cytoplasmic disk radiating from the nuclear surface to the cell cortex at the PPB plane (Figure 9A, Video S1). Later on, PLDα1-YFP was strongly accumulated in microtubule arrays of the mitotic spindle which was surrounded by cytoplasmic layer enriched with PLDα1-YFP (Figures 8B,C). Association of PLDα1-YFP with microtubules was documented by missing signal in the mitotic spindle occupied by chromosomes during metaphase and anaphase (Figures 8B–D). Starting with the segregation of sister chromatids and their pulling to the opposite spindle poles, PLDα1-YFP accumulated also in the central zone of the anaphase spindle (Figures 8C,D). Appearance of the early phragmoplast was connected with accumulation of PLDα1-YFP (Figure 8D). However, PLDα1-YFP was absent in the late phragmoplast mid-zone during cell plate formation in cytokinesis (Figure 8E). In addition, PLDα1-YFP was accumulated also in surrounding cytoplasm (phragmosome) enclosing cytokinetic apparatus in the center of partially vacuolated cells (Figures 8E, 9B, Video S2). As the late phragmoplast reached the cell periphery, PLDα1-YFP was associated with emerging cell plate in the central zone of the ring phragmoplast (Figure 9B, Video S2). Visual comparison of PLDα1-YFP protein level in cortical cytoplasm between dividing cells and neighboring non-dividing cells (Figures 8A,E) clearly indicated intracellular relocation and accumulation of PLDα1-YFP within and around mitotic microtubule arrays. It indicates potential cell cycle-dependent cooperation of PLDα1-YFP with microtubules in proliferating cells.

**Figure 10:**
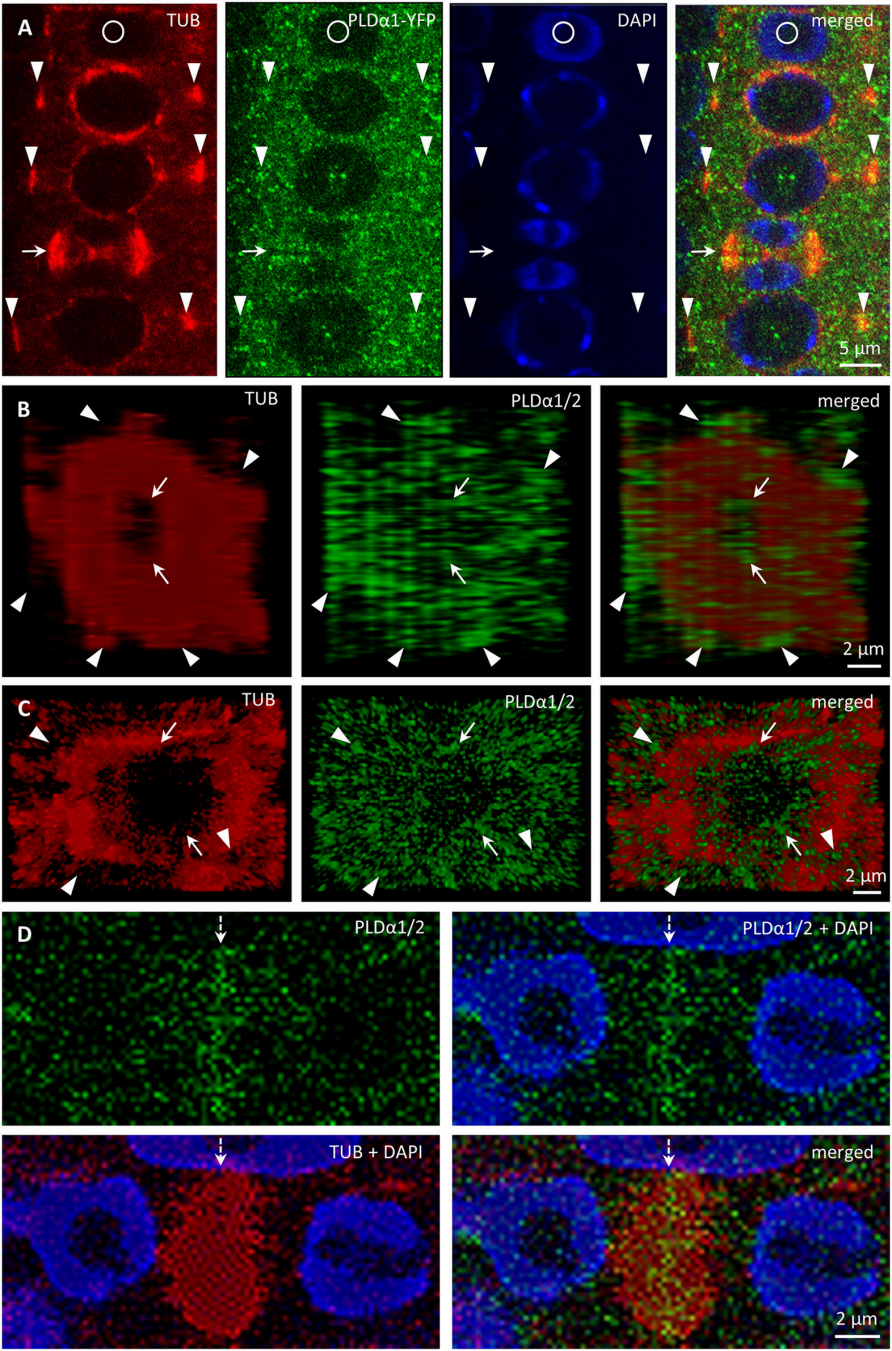
Immunofluorescence colocalization of microtubules and PLDα1–YFP during cell division in Arabidopsis root meristem cells of complemented *pldα1-1* mutant seedlings expressing PLDα1-YFP. **(A)** Colocalization of PLDα1-YFP with microtubules of PPB (arrowheads) and late phragmoplast (arrow). Note that circle indicates non-dividing cell. **(B)** Orthogonal projection of disk phragmoplast stage with the distribution of PLDα1-YFP at the leading (outer) edge (arrowheads) as well as at the trailing (inner) edge in the central part (arrows) of the phragmoplast. Still image of the orthogonal projection corresponds to Video S3. **(C)** Orthogonal projection of more advanced ring phragmoplast showing association of PLDα1-YFP with the leading edge (arrowheads) and the trailing edge (arrows) of the phragmoplast. Still image of the orthogonal projection corresponds to Video S4. **(D)** Accumulation of PLDα1-YFP in central part of the phragmoplast (arrow) at later stages of cytokinesis.

Association of PLDα1-YFP with mitotic microtubule arrays was confirmed using immunofluorescence colocalization of PLDα1-YFP and microtubules in root meristem cells of complemented *pldα1-1* mutant seedlings expressing *proPLDα1::PLDα1:YFP* construct. In non-dividing interphase or pre-mitotic cells PLDα1-YFP protein was localized in the cytoplasm, showing rather homogeneous distribution (Figure 10A). However, accumulation as well as partial association of PLDα1-YFP signal with microtubules of PPB, spindle and both early and late phragmoplast was observed in mitotic root cells (Figure 10A). Detailed analysis of PLDα1-YFP distribution during cytokinesis revealed its specific association with progressing phragmoplast. 3-D reconstruction, orthogonal projection and rotation of disk phragmoplast with initiated depolymerisation of microtubules in the central part showed predominant association of PLDα1-YFP with leading (outer) edge of the phragmoplast as well as with the trailing (inner) edge, which was created in the central part of the phragmoplast (Figure 10B, Video S3). Accumulation of PLDα1-YFP at the trailing edge of the late ring phragmoplast was more evident at later stages of the phragmoplast expansion (Figure 10C, Video S3). During phragmoplast enlargement to the cell edges and consolidation of newly formed daughter nuclei, PLDα1-YFP appeared to be more accumulated in the developing cell plate (Figure 10D). These results are consistent with PLDα1-YFP accumulation within and around mitotic microtubule arrays during cell division observed by live cell imaging (Figure 8).

Next, we employed immunofluorescence localization of tubulin in dividing and non-dividing interphase cells of primary roots of *pldα1-1* mutant to characterize possible involvement of PLDα1 on general microtubule organization (Figure S7). However, no obvious differences in the microtubule organization were observed during mitosis, cytokinesis or in non-dividing interphase cells of *pldα1-1* mutant in comparison to wild type Col-0 plants (Figure S7).

### Association of PLDα1-YFP with microtubules and CCVs and CCPs

In order to address the functional relationship between mitotic microtubules and PLDα1 in vesicular trafficking, we performed immunofluorescence localization of PLDα1-YFP with microtubules and CCVs and CCPs in dividing and non-dividing rhizodermal cells of complemented *pldα1-1* mutant seedlings expressing *proPLDα1::PLDα1:YFP* construct (Figure S8). In cytokinetic cells, PLDα1-YFP was associated with microtubules of the ring phragmoplast, while signal was less abundant in the phragmoplast central part. The distribution of clathrin signal in this zone was complementary to PLDα1-YFP distribution, with increased abundance in the central part and decreased abundance at the zone of phragmoplast microtubules (Figure S8A). Thus, the highest overlap of the clathrin and the PLDα1-YFP signal was observed at the trailing (inner) edge of the enlarging phragmoplast (Figure S8A).

**Figure 11:**
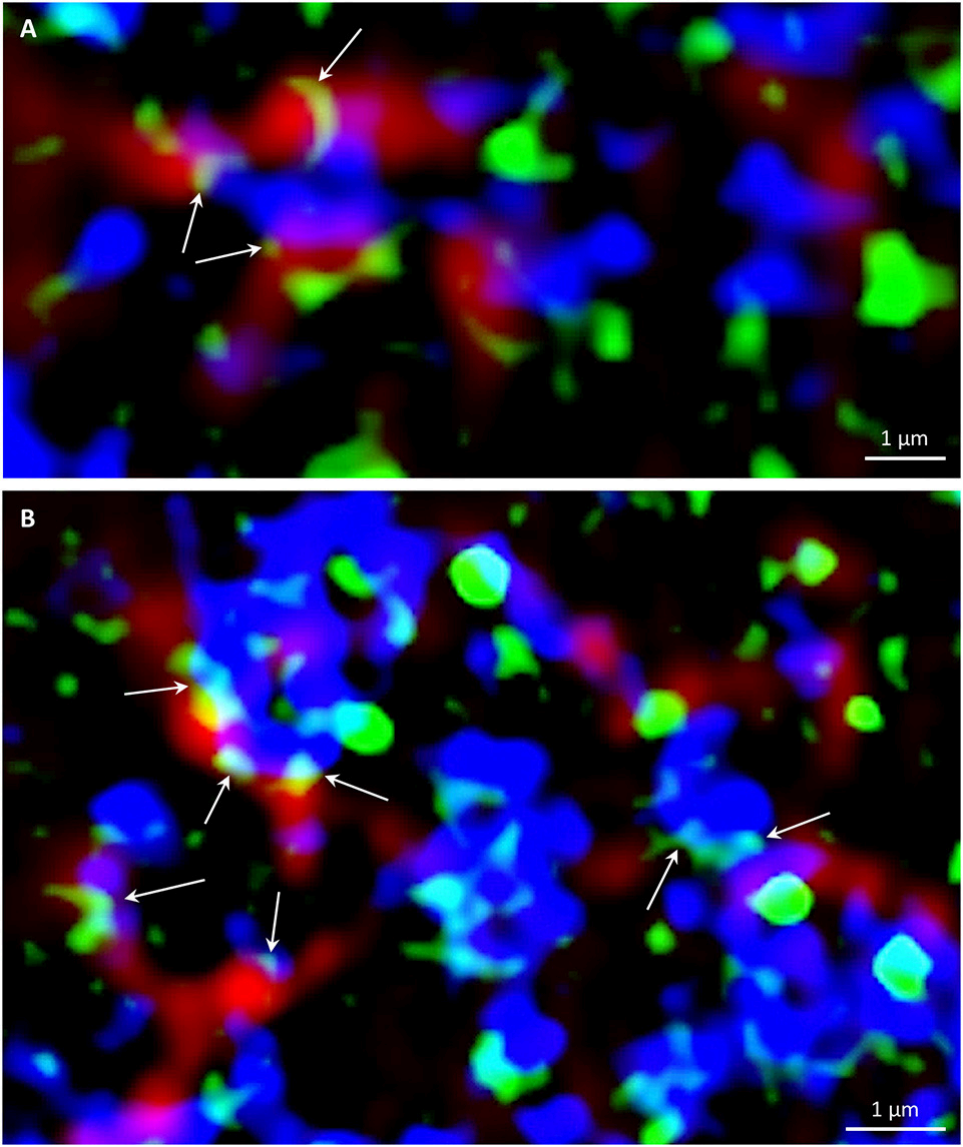
Super-resolved SIM immunofluorescence colocalization of cortical microtubules (red), PLDα1–YFP (green) and clathrin (blue) in Arabidopsis root cells of complemented *pldα1-1* mutant expressing PLDα1–YFP. Arrows indicate spot-like and ring-like structures of PLDα1–YFP in close contact with cortical microtubules and individual CCVs and CCPs **(A)**, or CCVs and CCPs arranged in clusters **(B)**.

In non-dividing rhizodermal cells cortical microtubules were bedecked with PLDα1-YFP closely associated or partially colocalizing with CCVs. PLDα1-YFP was localized in spot-like structures decorating surface of microtubules in close association or partial colocalization with CCVs (Figure S8B). For more detailed subcellular study of PLDα1 and microtubules involvement in vesicular trafficking we used super-resolved structural illumination microscopy (SIM). This analysis revealed association and partial colocalization of PLDα1-YFP with CCVs and CCPs in the close vicinity of cortical microtubules, in some cases creating spots and ring-like structures on their surface (Figure 11A). Association of PLDα1-YFP with CCVs and CCPs in the cytoplasm between cortical microtubules was observed in clathrin-rich clusters that were in close contact with cortical microtubules (Figure 11B). Quantitative analysis of the above colocalization studies of clathrin and microtubules following either SIM or CLSM documentation, showed positive colocalization in Col-0 rhizodermal cells (Pearson's coefficient *R* = 0,62) and no colocalization in *pldα1* mutant (Pearson's coefficient *R* = 0). In conclusion, these data document a complex pattern of PLDα1 subcellular localization and its functional relationship to microtubule arrays in both non-dividing and dividing cells of Arabidopsis plants. Combination of different advanced microscopy methods provided data supporting a possible mechanism of interactions between clathrin-dependent endocytosis and cortical (as well as mitotic) microtubules, through the stabilization function of the PLDα1.

**Figure 12:**
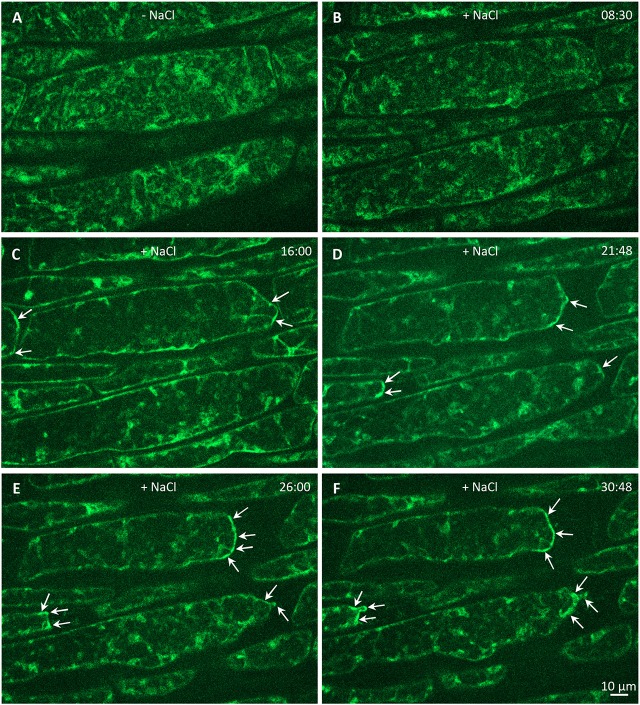
Effect of salt stress on relocalization of PLDα1-YFP (shown in artificial green color) driven by its native promoter in hypocotyl epidermal cells of complemented *pldα1-2* mutant. **(A)** Time-lapse imaging of PLDα1-YFP in hypocotyl epidermal cells under control conditions without salt and **(B–F)** during plasmolysis induced by 500 mM NaCl. The dynamic relocation and accumulation of PLDα1-YFP in plasmolyzed surface areas of detaching protoplasts is depicted by arrows. Time is indicated in min:sec.

Finally, we wanted to test whether stress factors such as high salinity can induce subcellular relocation of PLDα1-YFP in Arabidopsis cells. Indeed, PLDα1-YFP was relocated and accumulated in curved surface areas of plasmolysed protoplasts of hypocotyl cells treated with 500 mM NaCl (Figure 12). These data suggest a possible protective role of PLDα1 in these curved areas of retracting protoplasts detached from the cell wall during plasmolysis.

## Discussion

The most prominent phospholipid-hydrolyzing enzymes in plants are members of phospholipase D (PLD) family. *A. thaliana* PLDα1 and its product phosphatidic acid (PA) are closely associated with a wide range of cellular and physiological functions, such as regulation of stomatal closure and opening, cytoskeletal reorganization and stress signaling (Qin et al., 1997; Zhang et al., 2004; Bargmann and Munnik, 2006; Pleskot et al., 2013; Hong et al., 2016). Here we employed advanced microscopic methods to reveal precise developmental expression pattern and subcellular localization of PLDα1 in two rescued *pldα1* mutant lines.

ABA is an important hormone that regulates the adaptation of plants to various abiotic stresses (Danquah et al., 2014). It was shown by previous studies that *pldα1* mutant plants did not exhibit significant differences in morphological and developmental characteristics as compared to wild type plants (Fan et al., 1997; Zhang et al., 2012). However, PLDα1 controls proper water balance in plant responding to ABA hormone by stomatal closure which was impaired in *pldα1* knockout mutants (Zhang et al., 2004; Guo et al., 2012; Jiang et al., 2014). Here we transformed *pldα1-1* and *pldα1-2* mutant plants with *proPLDα::PLDα1-YFP* construct and we observed stomatal closure after ABA treatment similar to the wild type, suggesting functional complementation of both mutants by this construct.

### Developmental expression pattern and localization of PLDα1-YFP

A previous study assessing organ distribution of PLDα1 protein in Arabidopsis plants by immunoblotting analysis showed higher amounts of this protein in stems, flowers, roots, siliques, and old leaves. Moreover, the highest activity of PLDα1 was found in soluble fractions isolated from roots, flowers, and siliques (Fan et al., 1999). This study, however, lacked cellular resolution within these organs. In contrast, advanced microscopy imaging used in our experiments revealed high expression levels of PLDα1-YFP in the apical and lateral root cap cells. These findings were in agreement with absolute expression levels of PLDα1 transcript from Genevestigator transcriptomic data (Brady et al., 2007). High expression levels of PLDα1-YFP were found also in trichoblast cell files and in developing roots hairs suggesting its role during root hair development. These results are consistent with work of Potocký et al. (2014) reporting PA localization in the plasma membrane of tip-growing pollen tubes. In the aerial part of the plant we observed high PLDα1-YFP protein signal in pavement and stomata guard cells, which is again in accordance to Genevestigator transcriptomic data (Yang et al., 2008). On the other hand, and contrary to Genevestigator transcriptomic data (Marks et al., 2009), we observed high expression levels of PLDα1-YFP in developing trichomes. These results support the role of PLDα1 protein in cell developmental processes and polar cell growth.

### Cytoplasmic localization of PLDα1-YFP

The subcellular distribution of PLDα1 based on immunoblotting analyses of fractionated extracts of Arabidopsis leaves revealed the highest content at the plasma membrane, CCVs, intracellular membranes and mitochondria while only a small amount of protein was detected in nuclei (Fan et al., 1999).

Previously, it was reported that activation of PLD leads to rearrangement of cortical microtubules in suspension BY-2 cells (Dhonukshe et al., 2003). Later it was shown that PLDδ is the cortical microtubule-binding protein (Andreeva et al., 2009; Ho et al., 2009). Moreover, a current study revealed colocalization of the PLDδ with cortical microtubules near to the plasma membrane in the hypocotyl cells of Arabidopsis (Zhang et al., 2017b). Microtubule dynamics controls important processes such as mitosis, cytokinesis, cell elongation, and signal transduction (Wymer and Lloyd, 1996; Hashimoto and Kato, 2006; Jiang et al., 2014). Under normal conditions, knockout mutant *pldα1* showed no changes in microtubule organization and density as compared to wild type plants (Zhang et al., 2012). However, PLDα1 knockout in Arabidopsis leads to more severe disruption of cortical microtubules by inhibitors (Zhang et al., 2017a) or under salt stress conditions (Zhang et al., 2012). Furthermore, under the salt stress conditions PLDα1 is activated and produce PA which directs AtMAP65-1 to the plasma membrane, leading to microtubule stabilization and enhanced cell protection (Zhang et al., 2012; Pleskot et al., 2014).

PLDα1 is localized in both soluble and membrane fractions. It can translocate from cytosolic to membrane fractions and perform hydrolysis of membrane lipids under stress conditions (Wang et al., 2000; Hong et al., 2016). In our study we observed mainly cytoplasmic localization of PLDα1-YFP, sometimes in the vicinity of cortical microtubules, in non-dividing cells. By contrast, the localization of PLDδ which interacts with cortical microtubules was primarily restricted to the plasma membrane (Zhang et al., 2017b). With the entering of the cell to the mitosis PLDα1-YFP was enriched at mitotic microtubule arrays, namely PPB, microtubules of mitotic spindle in prophase, metaphase and anaphase, as well as to microtubules of the phragmoplast during cytokinesis. Although, colocalization between PLDα1 protein and microtubules was not previously observed in non-dividing protoplasts (Zhang et al., 2012), we detected accumulation of PLDα1-YFP at microtubule arrays during mitotic progression. These results were further confirmed using immunofluorescence colocalization of PLDα1-YFP and microtubules. PLDα1-YFP protein was slightly accumulated and partially colocalized with microtubules of preprophase band, spindle, and both early and late phragmoplast.

Multiple PLDs have been implicated to have redundant roles in ABA signaling and hyperosmotic stress. However, their mechanisms of action might be different. Single knockouts of either *PLDα1* or *PLD*δ cause the inhibition of ABA-induced stomatal closure while stomata of double mutant are almost completely insensitive to ABA (Zhang et al., 2004; Guo et al., 2012; Uraji et al., 2012; Hong et al., 2016). Moreover, PLDα1, through PA as an intermediate, promotes H_2_O_2_ production, whereas PLDδ mediates the response to H_2_O_2_ in the ABA signaling pathway. In this scenario PLDα1 can indirectly regulate PLDδ activity through ROS production (Guo et al., 2012; Hong et al., 2016). GhPLDα1 and H_2_O_2_ in the upland cotton (*Gossypium hirsutum*) are important components during the onset of the secondary cell wall thickening suggesting a putative role of PLDα1 in the vesicle trafficking (Tang and Liu, 2017). Furthermore, PLDα1 produced PA directs AtMAP65-1 to the plasma membrane and enhances its microtubule stabilizing activity, thus microtubules are stabilized and cell survival is enhanced under salt stress conditions (Zhang et al., 2012; Pleskot et al., 2014). Based on these findings, the question is posed whether PLDα1 can regulate mitotic progress alone or through PA, e.g., through the interaction with e.g., MAP65 proteins and/or PLDδ protein. More experiments should be performed to clarify the molecular mechanism by which PLDα1 affects mitotic microtubule arrays.

In mammals, PLD–PA signaling complexes regulate protein–membrane and membrane–cytoskeleton interactions, as well as vesicle budding and trafficking including exocytosis and endocytosis (McMahon and Gallop, 2005; Donaldson, 2009). Vesicle coating proteins, ARFs, Rho GTPases and soluble N-ethylmaleimide-sensitive factor attachment receptor (SNARE) proteins can interact with PLD and/or PA during vesicle formation at donor membranes, transport, docking, and fusion of vesicles with the target membranes (Chernomordik and Kozlov, 2003; Donaldson, 2009). In plants, there is some evidence that ARFs, Rho GTPases, SNARE proteins and PLDs are involved in vesicle targeting to the cell division plane, vesicle fusion, and cell plate biogenesis, although the exact roles of particular signaling proteins remains elusive (Zárský et al., 2009; El Kasmi et al., 2013; Smertenko et al., 2017).

Spo14 is phospholipase D of *Saccharomyces cerevisiae*, which specifically hydrolyzes phosphatidylcholine to generate choline and PA. Spo14p is localized to the cytoplasm of vegetative cells, however, it relocates to the spindle pole bodies and prospore membrane during sporulation (Rudge et al., 1998; Liu et al., 2007). PA produced by Spo14p is required to localize t-SNARE protein Spo20p to the prospore membrane (Nakanishi et al., 2006; Liu et al., 2007). As mentioned above, PLDα1 was enriched in the CCVs predicting its role in vesicular trafficking (Fan et al., 1999). Furthermore, AP180 (N-terminal homology domain clathrin-assembly) proteins and clathrins were identified as PA binding proteins in a previous proteomic study (McLoughlin et al., 2013). Similarly, epsin-like clathrin adaptor 1 binds PA under the negative membrane curvature stress in Arabidopsis (Putta et al., 2016). Furthermore, PLDα1 coimmunoprecipitates with Arabidopsis AP-2 complex and clathrin (Yamaoka et al., 2013), indicating that PLDα1 contributes to clathrin-mediated endocytosis. These data indicate that proteins involved in the clathrin-dependent endocytosis are potential targets of PLDα1-generated PA. Here we provide evidence for close associations of PLDα1-YFP with cortical and mitotic microtubules during cell division. It has been shown previously that the PPB region at the cell cortex possess a large number of CCPs and CCVs. Expected role of clathrin-mediated endocytosis in the PPB area is related to the regulated modification of the cell cortex by controlled removal of particular membrane proteins by endocytosis, being part of the cell division plane memory establishment (Karahara et al., 2009). Centrifugal expansion of the phragmoplast during cytokinesis is driven by microtubule polymerization with substantial microtubule stabilization by bundling at the leading edge of the phragmoplast (Murata et al., 2013). Phragmoplast microtubules are responsible for delivery of vesicles creating cell plate in the mid-zone region. Cell plate formation, however, is also based on removal of excess membrane and cell wall material. Endocytosis and membrane recycling thus play an indispensable role during cell plate expansion (van Oostende-Triplet et al., 2017). Endocytosis was implicated in the spatial restriction of syntaxin protein KNOLLE to the cell plate (Boutté et al., 2010) and in the removal of the cellulose synthase enzymes from the central part and their recycling to the peripheral growth zone of the cell plate (Miart et al., 2014). Consistently with these observations electron tomography analysis revealed a high density of CCPs and CCVs during the transformation of the tubulo-vesicular network to a planar fenestrated sheet during cell plate formation (Seguí-Simarro et al., 2004). CCPs and CCVs were mostly localized at the trailing (inner) edge of the enlarging phragmoplast. Internalization and recycling of material from the central part and its delivery to the leading edges of maturing cell plate were thus definitely connected to clathrin-dependent endocytosis (Boutté et al., 2010; Ito et al., 2012; Teh et al., 2013; Miart et al., 2014). Based on these findings and on our results we suggest that PLDα1 and its product PA might participate in the complex signaling network involved in the vesicle trafficking and membrane assembly during plant cytokinesis. Although the precise mechanism by which PLDα1 or PA are involved in these processes is unknown, our results indicate that PLDα1 localized on microtubule surface can potentially functions as molecular glue for CCPs and CCVs associated with microtubules. This is further corroborated by the observation that microtubule-colocalized clathrin structures are quantitatively absent in the *pldα1* mutant.

In animal literature it is suggested that PLD and PA participate in vesicle formation at various cellular membranes. One possible mechanism is that physiochemical properties of PLD and PA as well as their protein-protein and lipid-protein interactions (e.g., with dynamin, COPI, kinesin, ARF, small GTPases, phosphatases, kinases, and phosphoinositols) might regulate such vesicle formation (Manifava et al., 2001; Roth, 2008; Brito de Souza et al., 2014). More physiological and functional studies will be needed to prove this concept experimentally. But complex pattern of PLDα1 developmental expression, subcellular localization, and its close association with cortical and mitotic microtubules in Arabidopsis documented here by advanced microscopy methods substantially contribute to this scenario and will promote further research in this topic.

## Author contributions

DN, PV, MO, OŠ, and JC: Conducted experiments; DN, MO, and OŠ: made image post acquisition analyses; GK: Helped with quantitative evaluations and data interpretation; PV, DN, MO, OŠ, and JŠ: Wrote the manuscript with input from all co-authors; JŠ: Proposed experiments and supervised this study, participated on data interpretation and finalized manuscript.

### Conflict of interest statement

The authors declare that the research was conducted in the absence of any commercial or financial relationships that could be construed as a potential conflict of interest.

## Supplementary material

Figure S1 Comparison of PLDα1-YFP localization in different aerial organs and tissues of *pldα1-1* **(A–E)** and *pldα1-2* **(F–J)** mutants stably expressing PLDα1-YFP driven by its own promoter: epidermis of first true leaf **(A,F)**, epidermal cells of hypocotyl **(B,G)**, epidermal cells of cotyledon petiole **(C,H)**, epidermal cells of cotyledon **(D,I)** and entire 5 days-old seedling **(E,J)**.

Figure S2 PLDα1-YFP localization in different tissues of the root tip of rescued *pldα1-1* mutant stably transformed with *proPLDα1::PLDα1:YFP* construct by light-sheet fluorescence microscopy. **(A)** Localization of PLDα1-YFP in cells of the root tip counterstained by propidium iodide and merged image. Profiles at different positions of the root tip are visualized in orthogonal projections of radial root sections in **(B)**.

Figure S3 PLDα1-YFP localization in different tissues of the root tip of rescued *pldα1-2* mutant stably transformed with *proPLDα1::PLDα1:YFP* construct by light-sheet fluorescence microscopy. **(A)** Localization of PLDα1-YFP in cells of the root tip counterstained by propidium iodide and merged image. Profiles at different positions of the root tip are visualized in orthogonal projections of radial root sections in **(B)**.

Figure S4 PLDα1-YFP localization in trichoblast (labeled as T) and atrichoblast (labeled as A) rhizodermis cell files of the root tip of rescued *pldα1-1* mutant stably transformed with *proPLDα1::PLDα1:YFP* construct by light-sheet fluorescence microscopy. Localization of PLDα1-YFP, propidium iodide and merged image of the root transition zone in longitudinal **(A)** and transversal **(B)** root projections.

Figure S5 PLDα1-YFP localization in trichoblast (labeled as T) and atrichoblast (labeled as A) rhizodermis cell files of the root tip of rescued *pldα1-2* mutant stably transformed with *proPLDα1::PLDα1:YFP* construct by light-sheet fluorescence microscopy. Localization of PLDα1-YFP, propidium iodide and merged image of the root transition zone in longitudinal **(A)** and transversal **(B)** root projections.

Figure S6 Immunofluorescence localization of PLDα1 protein in Arabidopsis root meristem cells of wild type Col-0 seedlings showing homogeneous distribution of PLDα1 in the cytoplasm.

Figure S7 Organization of microtubule arrays in dividing cells of root meristem in *pldα1-1* mutant in comparison to wild type Col-0. Arrowheads indicate PPBs, red arrows mitotic spindles and white arrows phragmoplasts. Immunofluorescence localization of microtubules with confocal microscopy, nuclei are counterstained with DAPI.

Figure S8 Immunofluorescence colocalization of microtubules with PLDα1–YFP and clathrin in Arabidopsis root cells of complemented *pldα1-1* mutant expressing PLDα1–YFP. **(A)** Colocalization of microtubules (green), PLDα1–YFP (red), and clathrin (blue) in late phragmoplast of root meristematic cell during the cytokinesis. **(B)** Colocalization of cortical microtubules (green), PLDα1–YFP (blue) and clathrin (red) in interphase root cell. Boxed areas in **(B)** are magnified in **(C)**. Arrows indicate colocalization of PLDα1–YFP with clathrin in association with cortical microtubules.

Video S1 3-D rendering of leaf epidermal petiole cell at the pre-prophase stage of cell division with established PPB and localization of PLDα1-YFP.

Video S2 3-D rendering of leaf epidermal petiole cell at the cytokinesis with ring phragmoplast and localization of PLDα1-YFP.

Video S3 3-D rendering of early disk phragmoplast in root meristematic cell at the cytokinesis with localization of PLDα1-YFP.

Video S4 3-D rendering of late ring phragmoplast in root meristematic cell at the cytokinesis with localization of PLDα1-YFP.
