# Supplementary material for: Gene Expression Pattern and Protein Localization of Arabidopsis Phospholipase D Alpha 1 Revealed by Advanced Light-Sheet and Super-Resolution Microscopy
Source: Front Plant Sci. 2018 Mar 21;9:371. doi: 10.3389/fpls.2018.00371 (PMC5877115; doi:10.3389/fpls.2018.00371)
Supplement: Figure S1 — Comparison of PLDα1-YFP localization in different aerial organs and tissues of pldα1-1 (A–E) and pldα1-2 (F–J) mutants stably expressing PLDα1-YFP driven by its own promoter: epidermis of first true leaf (A,F), epidermal cells of hypocotyl (B,G), epidermal cells of cotyledon petiole (C,H), epidermal cells of cotyledon (D,I) and entire 5 days-old seedling (E,J). [file Presentation1.pdf]

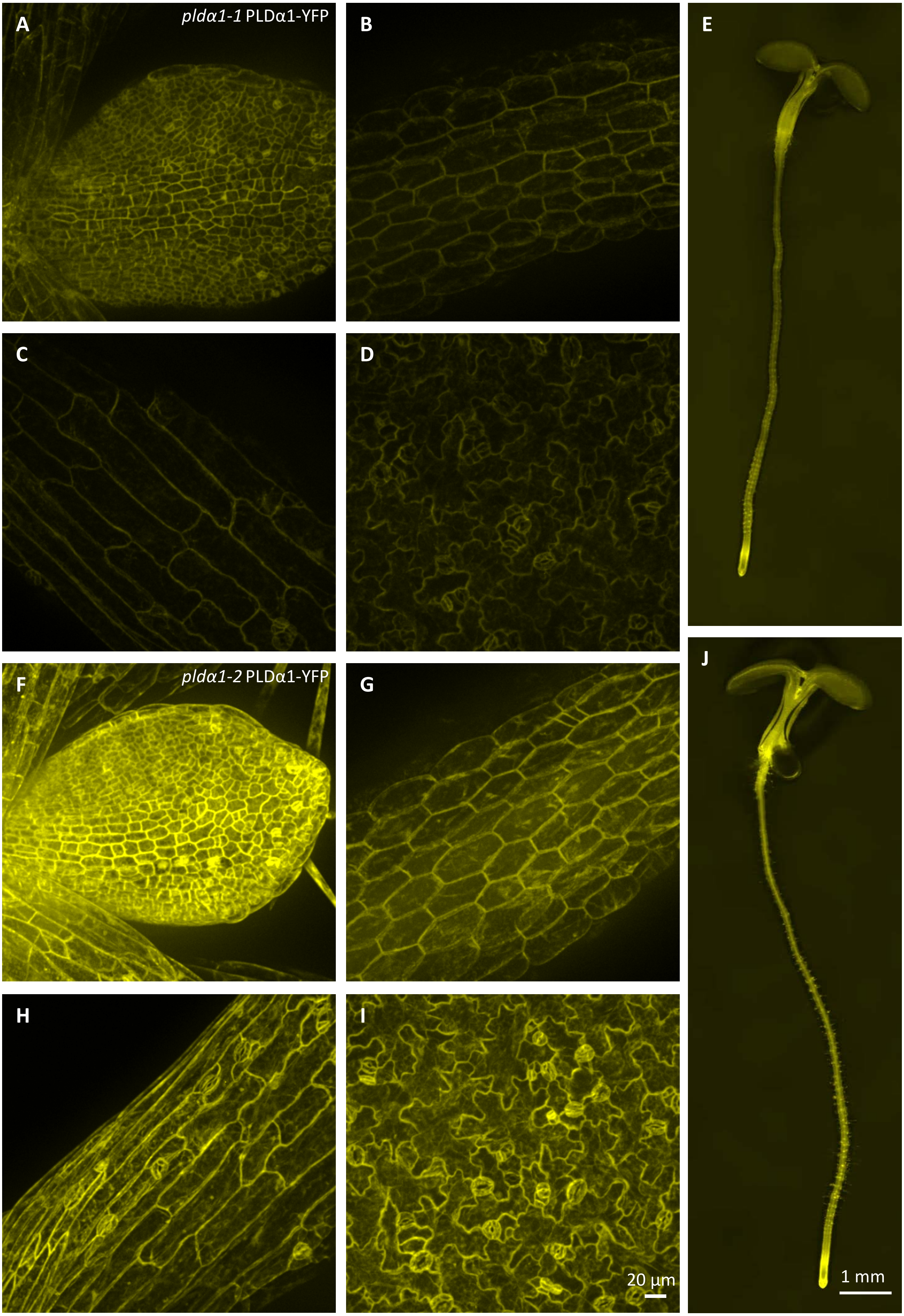

Figure S1. Comparison of PLDα1-YFP localization in different aerial organs and tissues of *pldα1-1* **(A-E)** and *pldα1-2* **(F-J)** mutants stably expressing PLDα1-YFP driven by its own promoter: epidermis of first true leaf **(A, F)**, epidermal cells of hypocotyl **(B, G)**, epidermal cells of cotyledon petiole **(C, H)**, epidermal cells of cotyledon **(D, I)** and entire 5 days-old seedling **(E, J)**.

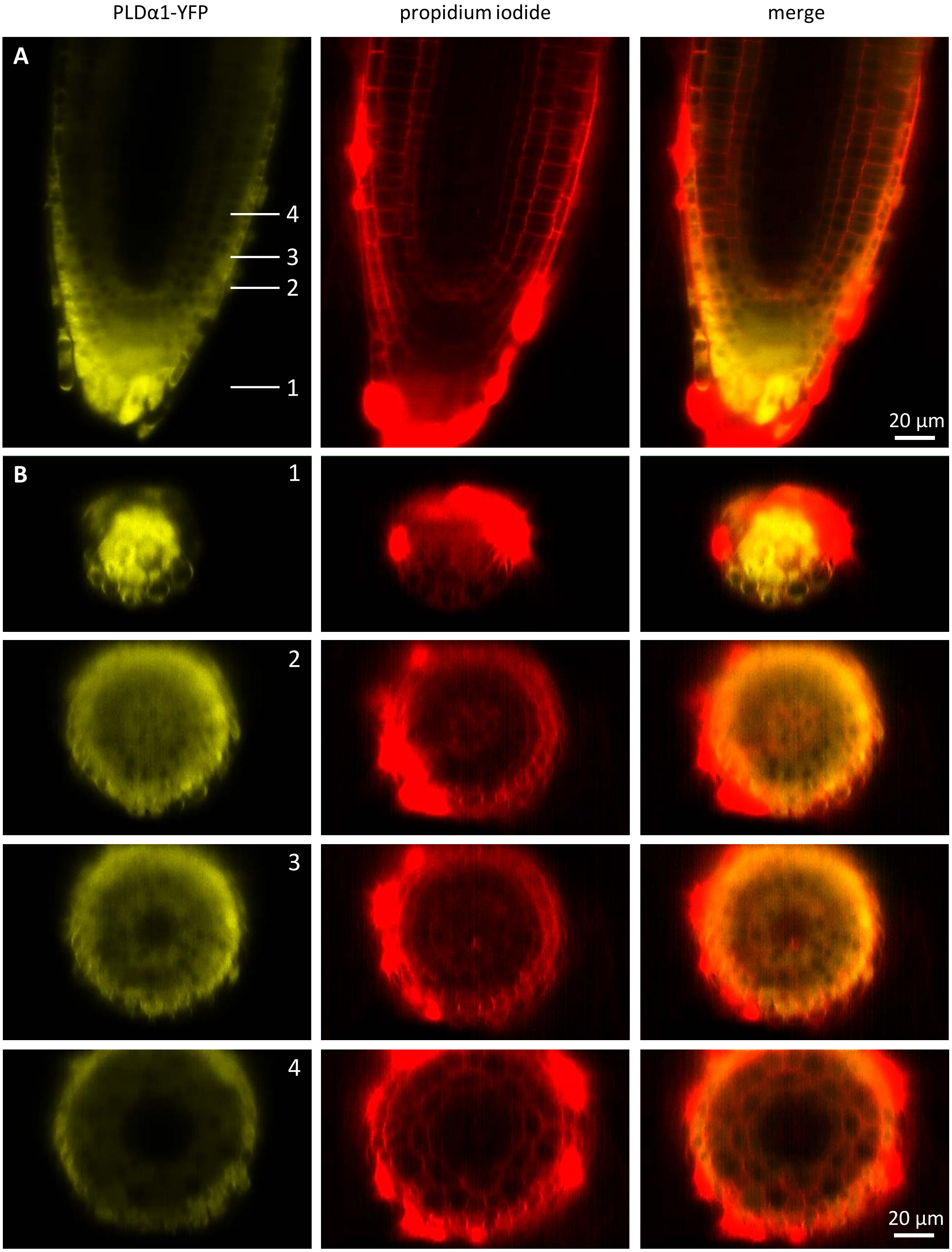

Figure S2. PLDα1-YFP localization in different tissues of the root tip of rescued *pldα1-1* mutant stably transformed with *proPLDα1::PLDα1:YFP* construct by light-sheet fluorescence microscopy. **(A)** Localization of PLDα1-YFP in cells of the root tip counterstained by propidium iodide and merged image. Profiles at different positions of the root tip are visualized in orthogonal projections of radial root sections in **(B)**.

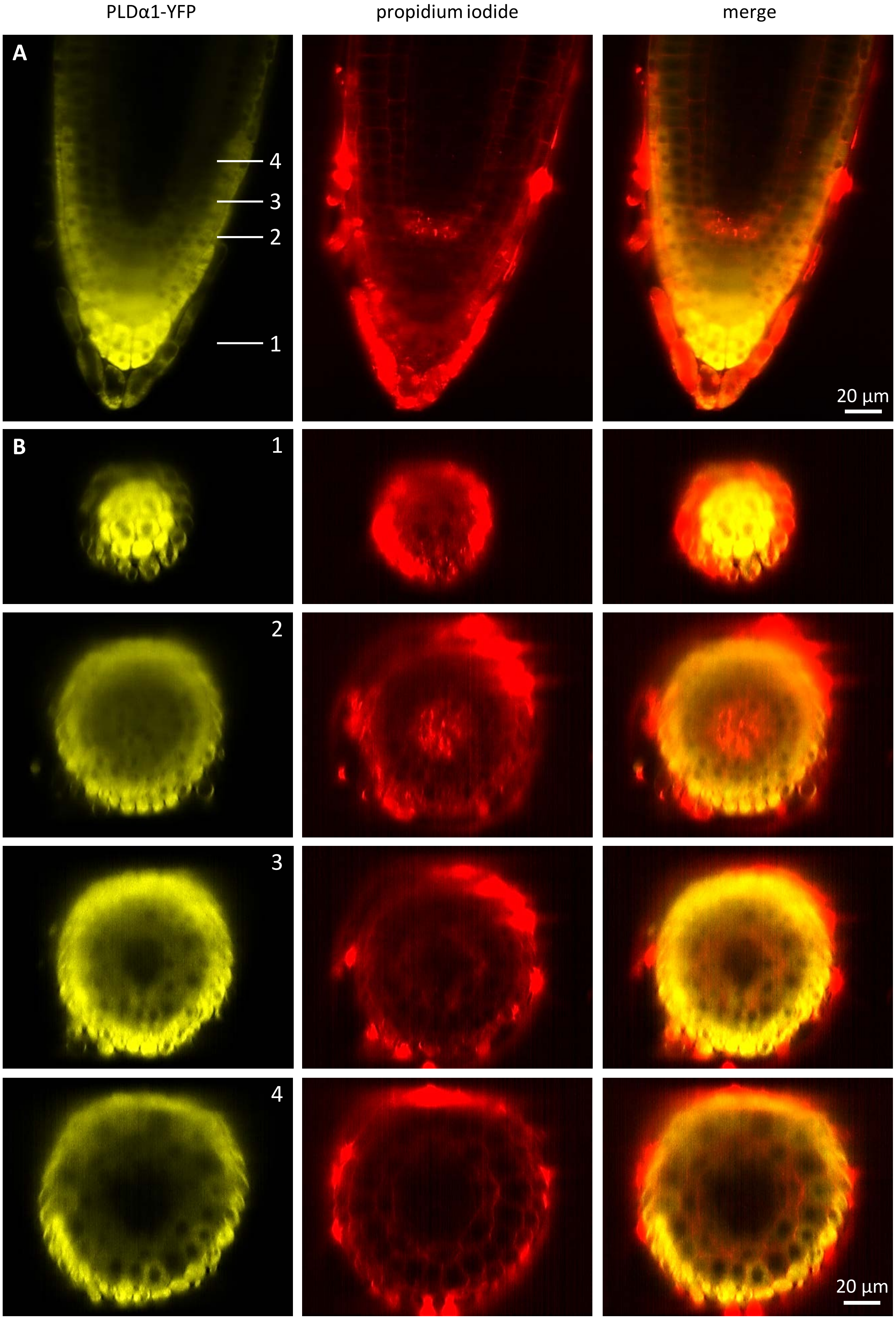

Figure S3. PLDα1-YFP localization in different tissues of the root tip of rescued *pldα1-2* mutant stably transformed with *proPLDα1::PLDα1:YFP* construct by light-sheet fluorescence microscopy. **(A)** Localization of PLDα1-YFP in cells of the root tip counterstained by propidium iodide and merged image. Profiles at different positions of the root tip are visualized in orthogonal projections of radial root sections in **(B)**.

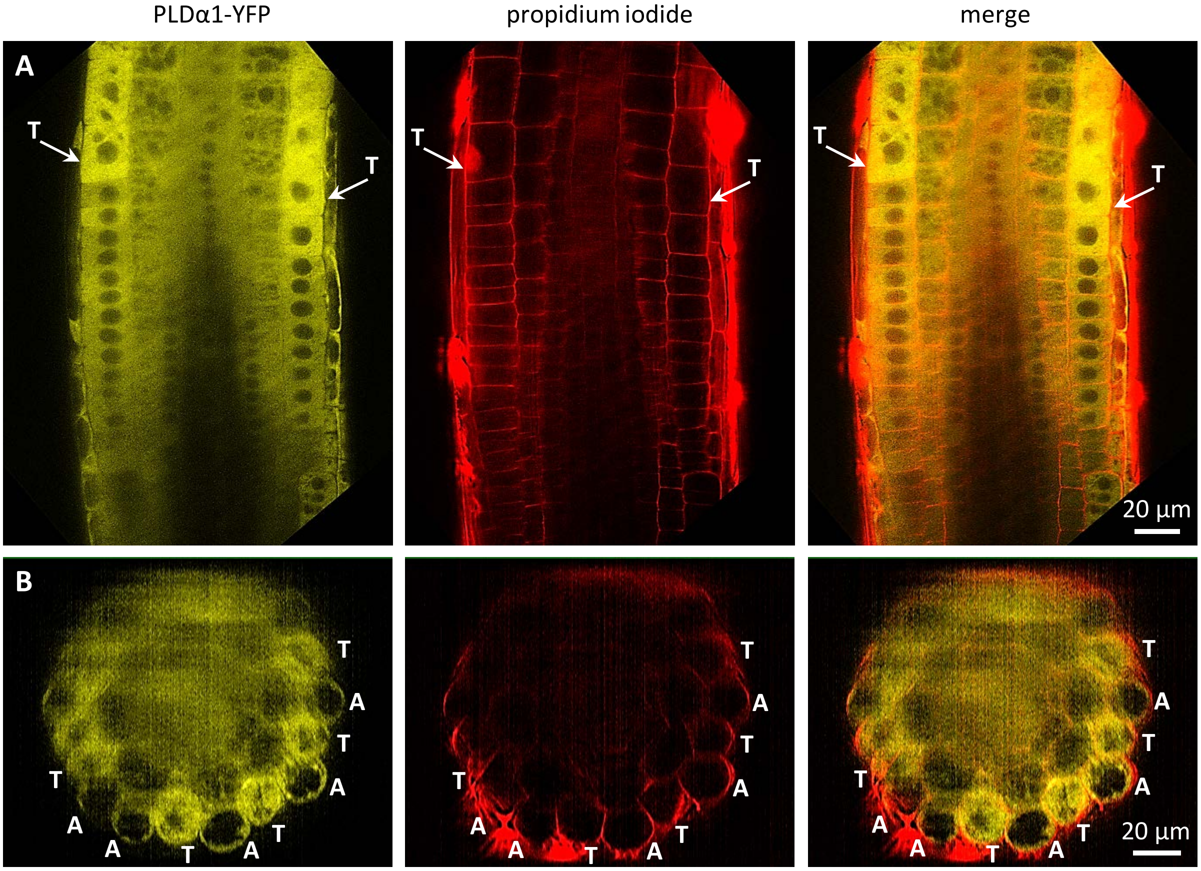

Figure S4. PLDα1-YFP localization in trichoblast (labelled as T) and atrichoblast (labelled as A) rhizodermis cell files of the root tip of rescued *pldα1-1* mutant stably transformed with *proPLDα1::PLDα1:YFP* construct by light-sheet fluorescence microscopy. Localization of PLDα1-YFP, propidium iodide and merged image of the root transition zone in longitudinal **(A)** and transversal **(B)** root projections.

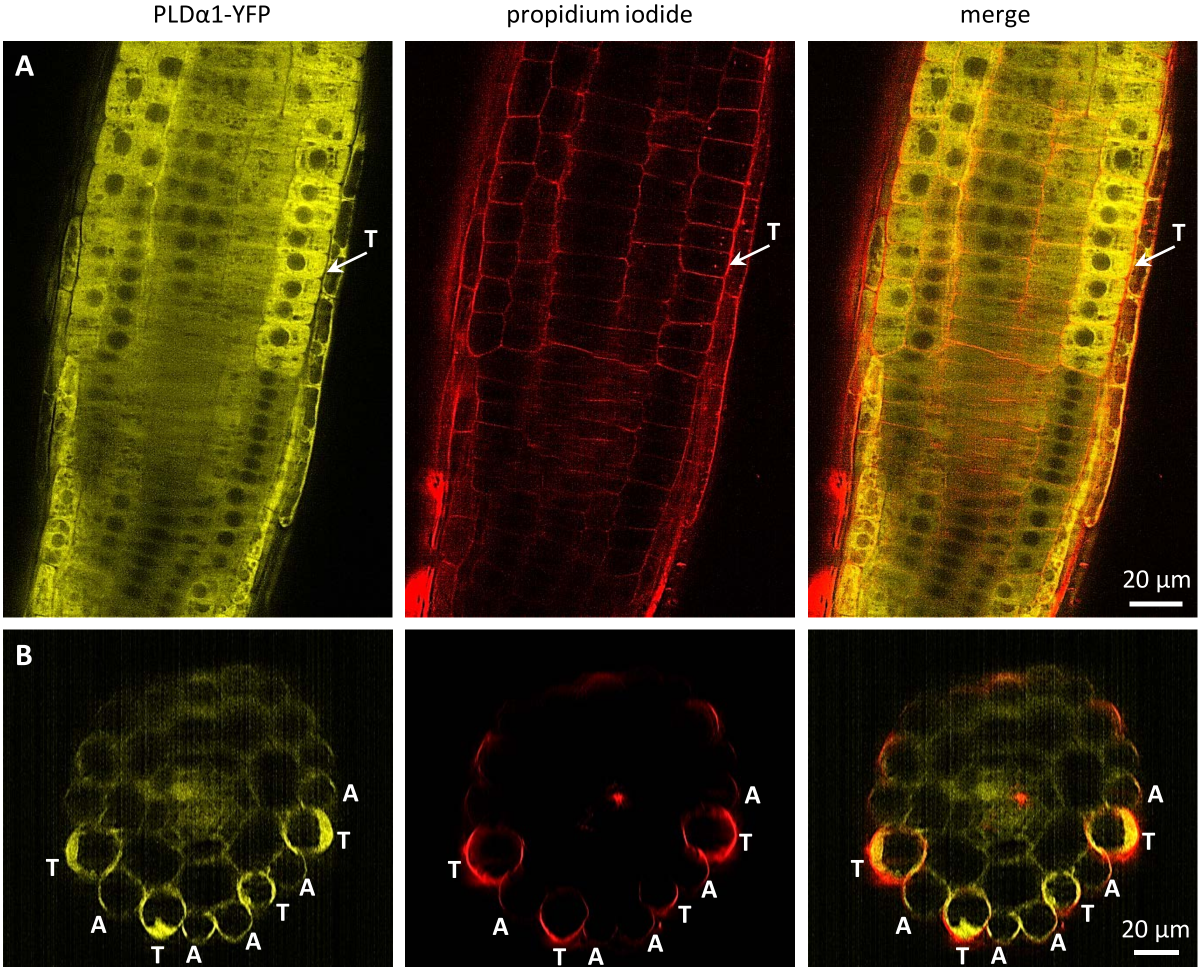

Figure S5. PLDα1-YFP localization in trichoblast (labelled as T) and atrichoblast (labelled as A) rhizodermis cell files of the root tip of rescued *pldα1-2* mutant stably transformed with *proPLDα1::PLDα1:YFP* construct by light-sheet fluorescence microscopy. Localization of PLDα1-YFP, propidium iodide and merged image of the root transition zone in longitudinal **(A)** and transversal **(B)** root projections.

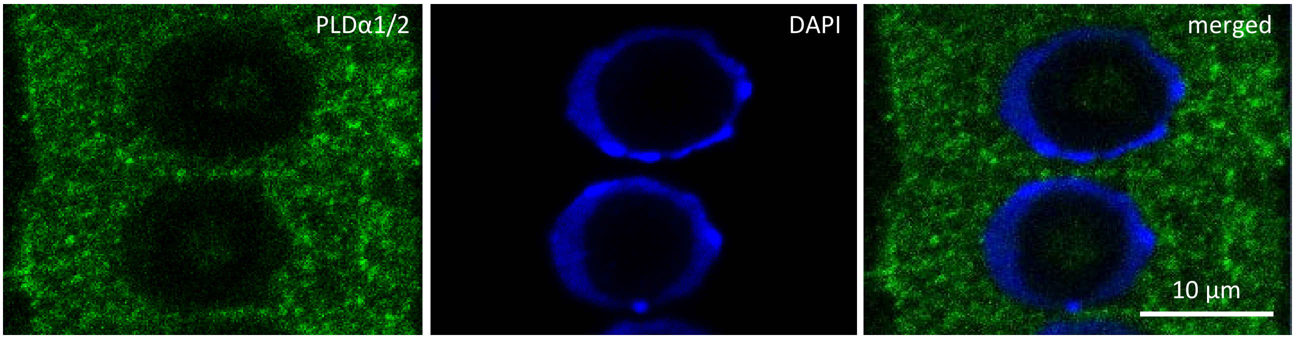

Figure S6. Immunofluorescence localization of PLDα1 protein in Arabidopsis root meristem cells of wild type Col-0 seedlings showing homogenous distribution of PLDα1 in the cytoplasm.

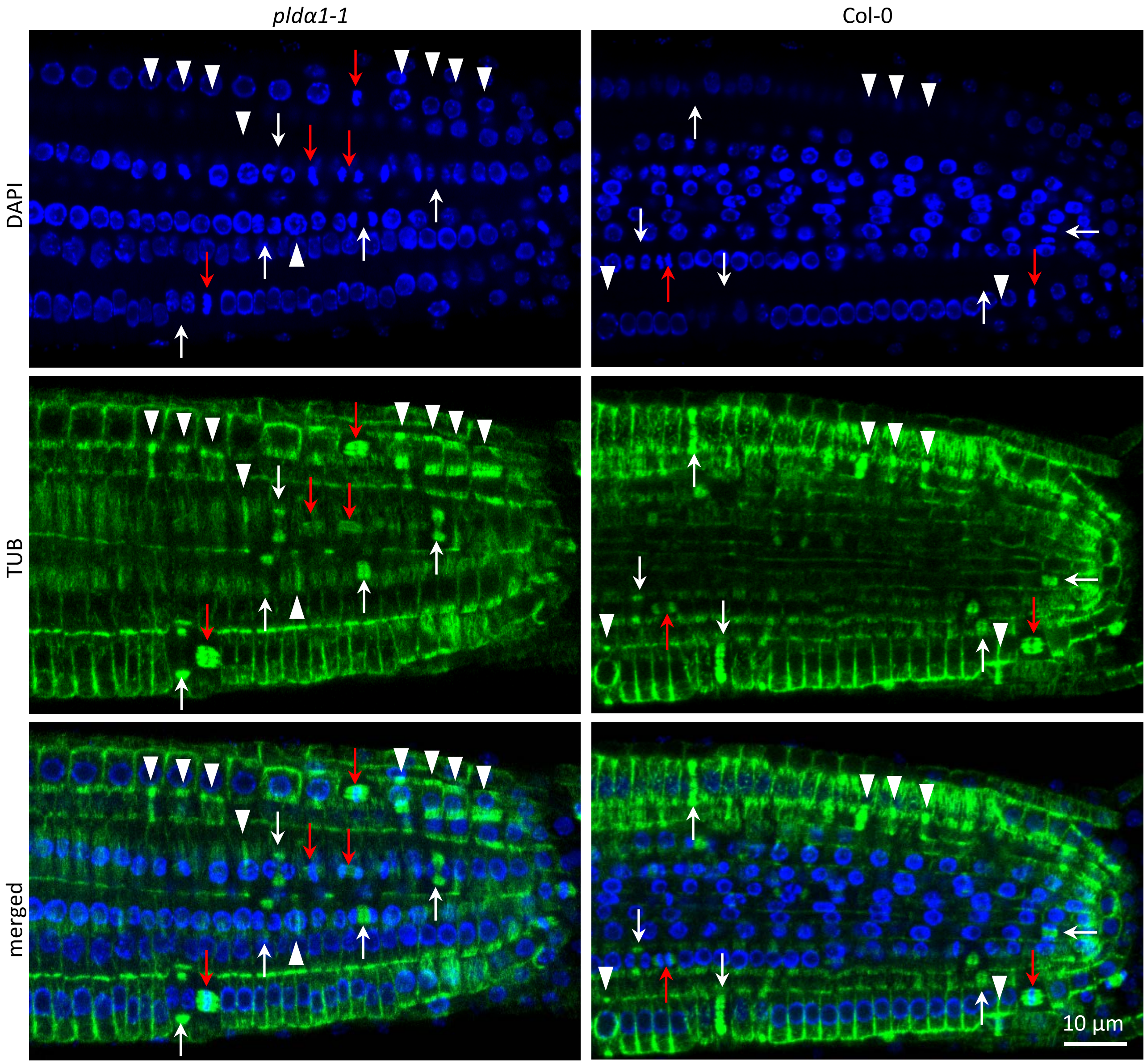

Figure S7. Organization of microtubule arrays in dividing cells of root meristem in *pldα1-1* mutant in comparison to wild type Col-0. Arrowheads indicate PPBs, red arrows mitotic spindles and white arrows phragmoplasts. Immunofluorescence localization of microtubules with confocal microscopy, nuclei are counterstained with DAPI.

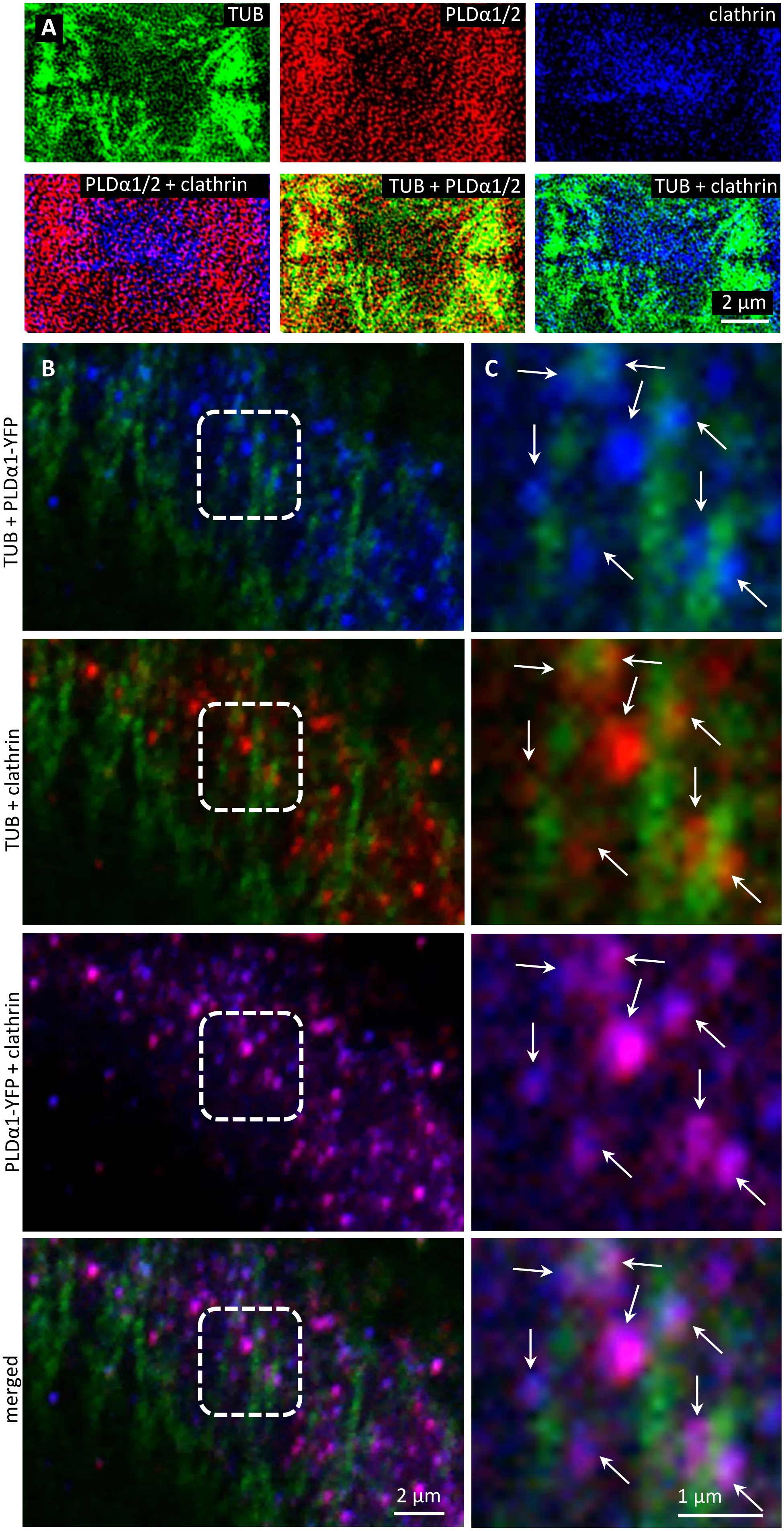

Figure S8. Immunofluorescence colocalization of microtubules with PLDα1–YFP and clathrin in Arabidopsis root cells of complemented *pldα1-1* mutant expressing PLDα1–YFP. **(A)** Colocalization of microtubules (green), PLDα1–YFP (red) and clathrin (blue) in late phragmoplast of root meristematic cell during the cytokinesis. **(B)** Colocalization of cortical microtubules (green), PLDα1–YFP (blue) and clathrin (red) in interphase root cell. Boxed areas in **(B)** are magnified in **(C)**. Arrows indicate colocalization of PLDα1–YFP with clathrin in association with cortical microtubules.
